# The oscillation of mitotic kinase governs cell cycle latches in mammalian cells

**DOI:** 10.1242/jcs.261364

**Published:** 2024-02-13

**Authors:** Calin-Mihai Dragoi, Ekjot Kaur, Alexis R. Barr, John J. Tyson, Béla Novák

**Affiliations:** ^1^ Department of Biochemistry, University of Oxford, South Parks Road, Oxford OX1 3QU, UK; ^2^ MRC London Institute of Medical Sciences, Hammersmith Hospital Campus, Du Cane Road, London W12 0NN, UK; ^3^ Institute of Clinical Sciences, Imperial College London, Du Cane Road, London W12 0NN, UK; ^4^ Department of Biological Sciences, Virginia Tech, Blacksburg, VA 24061, USA

**Keywords:** Biochemical switches, Bistability, Endocycles, Hysteresis, Size control

## Abstract

The mammalian cell cycle alternates between two phases – S-G2-M with high levels of A- and B-type cyclins (CycA and CycB, respectively) bound to cyclin-dependent kinases (CDKs), and G1 with persistent degradation of CycA and CycB by an activated anaphase promoting complex/cyclosome (APC/C) bound to Cdh1 (also known as FZR1 in mammals; denoted APC/C:Cdh1). Because CDKs phosphorylate and inactivate Cdh1, these two phases are mutually exclusive. This ‘toggle switch’ is flipped from G1 to S by cyclin-E bound to a CDK (CycE:CDK), which is not degraded by APC/C:Cdh1, and from M to G1 by Cdc20-bound APC/C (APC/C:Cdc20), which is not inactivated by CycA:CDK or CycB:CDK. After flipping the switch, cyclin E is degraded and APC/C:Cdc20 is inactivated. Combining mathematical modelling with single-cell timelapse imaging, we show that dysregulation of CycB:CDK disrupts strict alternation of the G1-S and M-G1 switches. Inhibition of CycB:CDK results in Cdc20-independent Cdh1 ‘endocycles’, and sustained activity of CycB:CDK drives Cdh1-independent Cdc20 endocycles. Our model provides a mechanistic explanation for how whole-genome doubling can arise, a common event in tumorigenesis that can drive tumour evolution.

## Teaser

**Summary:** Combining modelling and experimentation, we propose a latching-gate mechanism for strict alternation of DNA replication and mitosis in human cells and show that, if the latches are broken by mutation, then endocycles ensue.

## INTRODUCTION

**Fig. 1.**
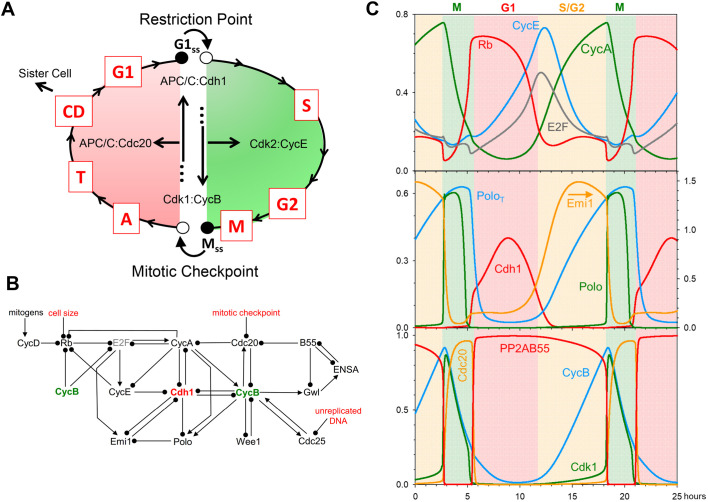
**A model of mammalian cell cycle controls.** (A) Conceptual framework. A newborn cell arrests in G1 phase (unreplicated chromosomes) at a stable steady state, denoted by the black circle labelled G1_ss_. Growth factors, integrated at the restriction point (RP), destabilize G1_ss_ (white circle) and induce the cell to enter S/G2/M phase (green), replicating its chromosomes and eventually arresting in mitosis at a different stable steady state, denoted by the black circle labelled M_ss_, while the replicated chromosomes are coming into alignment on the mitotic spindle. When the spindle is properly assembled and all chromosomes are properly aligned, the mitotic checkpoint is lifted, destabilizing M (black circle to white circle transition) and allowing the cell to exit mitosis (red phase: M→A→T), divide (CD) and return to the G1 stable state. These events are coordinated by a complex protein interaction network, whose principal components are displayed inside the cycle. (B) An influence diagram summarizing mammalian cell cycle controls. Arrowheads indicate ‘activation’ or ‘synthesis’; black dots indicate ‘inactivation’ or ‘degradation’. Cdh1 and CycB play central roles in the control system. At the G1 steady state, Cdh1 and Rb are active, E2F is inactive, the levels of the cyclins (CycA, CycB, CycE and CycD) are low, as are those of Emi1 and Polo. At the M steady state, Cdh1 is inactive, and CycA, CycB and Polo are active. This diagram is converted into a set of nonlinear ordinary differential equations in the Materials and Methods. (C) Limit-cycle oscillations of the model when all checkpoints are removed. The model ODEs are simulated numerically for the parameter values given in Table S1, and selected variables are plotted as functions of time (in hours). The phases of the cell cycle are colour coded: G1 (pink), S/G2 (yellow) and M (green). Notice that Rb and Cdh1 activities are high in G1 phase, CycE and E2F activities peak at the G1/S transition, Emi1 and CycA are high in G2 phase, ‘Cdk1 activity’ (i.e. active CycB:Cdk1) and Polo peak as the simulated cell enters mitosis, and Cdc20 peaks as the cell exits mitosis and returns to G1 phase. Meanwhile, PP2A:B55 activity is high throughout G1/S/G2 and low only when Cdk1 activity is high. Because no checkpoint controls are operational in this simulation, the cell cycle time-courses do not pause at the stable steady states (G1 and M) in A. In the middle panel, arrows indicate the corresponding y-axis for dynamic variables.

The eukaryotic cell cycle is the repetitive process of DNA synthesis (chromosome replication, S), metaphase (alignment of the replicated chromosomes on the mitotic spindle, M), anaphase (separation of the sister chromatids to opposite poles of the spindle, A), telophase (formation of daughter nuclei, each containing a full complement of unreplicated chromosomes, T) and cell division (separation into two, genetically identical daughter cells, CD; Fig. 1A). This cycle of DNA replication and chromosome partitioning runs in parallel to cell growth, whereby all other essential components of the cell (proteins, lipids, polysaccharides and organelles) are amplified and divided more-or-less evenly between the newborn daughter cells. The growth and division processes are balanced, in the long run, so that a proliferating cell population maintains a stable size and DNA distribution (Morgan, 2007).

Eukaryotic cells coordinate growth and division at ‘checkpoints’ – here defined as pauses in cell cycle progression lasting until specific physiological conditions are satisfied. Most somatic cells in animals arrest soon after birth (in G1 phase of the cycle, with unreplicated chromosomes) in a stable state, known as quiescence. To re-enter a new round of growth and division, cells must first pass the restriction point (RP). To pass the RP and progress into S-phase (DNA replication), a cell must receive ‘permission’ in the form of extracellular growth factors that disengage the ‘brakes’ holding the cell prior to the RP (Pardee, 1974). After passing the RP, the cell replicates its DNA and enters mitosis. A second crucial checkpoint, the mitotic checkpoint (MC), arrests the cell in mitosis until all the replicated chromosomes are properly aligned on the mitotic spindle (Musacchio, 2015). Then, and only then, does the cell receive permission to initiate anaphase and partition the sister chromatids to daughter nuclei.

These checkpoints and transitions are implemented by an exceedingly complex network of interacting genes and proteins (Kohn, 1999). In earlier publications (Chen et al., 2000; Novak and Tyson, 2022; Tyson and Novak, 2008), we have studied this network in detail for budding yeast cells (*Saccharomyces cerevisiae*). We proposed that – at its core – the cell cycle is an alternation between two fundamental phases: G1 (cells not committed to DNA replication and cell division) and S/G2/M (cells in progress toward mitosis and cell division). Each phase is attracted to a stable steady state of the underlying molecular regulatory network; let us denote them G1_ss_ and M_ss_. S/G2/M phase is characterized by rising activity of B-type cyclins (generically denoted as CycB; Clb1–Clb6 in yeast) bound to cyclin-dependent kinases (CDKs) (denoted Clb1–Clb6:CDK), driving S phase and entry into mitosis. S/G2/M phase ultimately terminates at M_ss_, a stable steady state characterized by high CDK activity and negligible activities of the CDK ‘antagonists’ that are prevalent in G1 phase. The uncommitted phase is characterized by a stable steady state, G1_ss_, of low CDK activity and high antagonist activities.

In budding yeast, the principal CDK antagonists are (1) the anaphase-promoting complex/cyclosome (APC/C), which promotes polyubiquitylation of five of the B-type cyclins (Clb1–Clb5) and their subsequent degradation by proteasomes, and (2) cyclin-dependent kinase inhibitors (CKIs), which bind to and inhibit the B-type CDKs (Nasmyth, 1996). In G1 phase, APC/C activity is directed towards Clb1–Clb5 by a targeting subunit called Cdh1 (known as FZR1 in mammals; APC/C bound to Cdh1 is denoted APC/C:Cdh1). Hence, we characterize G1_ss_ as a steady state with high activities of both APC/C:Cdh1 and CKIs. The B-type CDKs and their antagonists are mutually inhibitory – not only do the antagonists suppress CDK activity, but the CDKs phosphorylate both Cdh1 (phosphorylated Cdh1 is inactive) and CKIs [phosphorylated CKIs are rapidly degraded by the Skp/Cullin/F-box (SCF)-mediated polyubiquitylation pathway] (Nasmyth, 1996). The mutual inhibition between B-type CDKs and their antagonists is the source of the coexisting, stable steady states (M_ss_ and G1_ss_) of the underlying cell cycle control system in yeast (Chen et al., 2000; Novak and Tyson, 2022; Tyson and Novak, 2008).

Although we originally presented this concept of cell cycle regulation for budding yeast, it is applicable to eukaryotic organisms in general, because B-type CDKs are a universal feature of entry into mitosis (Nurse, 1990), and their opposition by APC/C:Cdh1 and stoichiometric CKIs is a universal feature of G1 arrest in eukaryotic cells. In this paper, we focus on the control system in mammalian cells. As suggested by Fig. 1A, the coexisting stable steady states (G1_ss_ and M_ss_) of the underlying bistable switch force the cell to follow a distinctive loop of cell-cycle events governed by two characteristic transitions: from G1 into S/G2/M as the RP is lifted, and from M into A/T/CD/G1 as the MC is lifted (Fig. 1A). At these transitions, the cell executes the two crucial events of the chromosome cycle: (1) passing from G1 into S/G2/M, during which the chromosomes in the cell are replicated and brought into alignment by the mitotic spindle, and (2) passing from M into A/T/CD/G1, when the sister chromatids are partitioned to two daughter cells.

These two transitions are fundamentally irreversible because of a ‘latching’ property of the bistable switch. At RP, the G1 steady state becomes unstable (denoted as the black circle to white circle transition in Fig. 1A), and the cell enters S/G2/M by upregulating cyclin E bound to Cdk2 (CycE:Cdk2), which promotes the rise of cyclin A bound to Cdk2 (CycA:Cdk2; involved in DNA replication) and CycB:Cdk1 (mitotic CDK activity). As the levels of CycA- and CycB-dependent kinases rise, CycE is phosphorylated and degraded by the SCF pathway (negative feedback) (Clurman et al., 1996). As CycE-dependent kinase activity drops, the control system is captured by the stable steady state M_ss_ (black circle in Fig. 1A). At RP, the ‘G1 gate’ is opened and CycE pushes the cell into S/G2/M. The negative feedback loop acts as a ‘spring’ to pull the gate closed, and it ‘latches’ at the stable M state. For the cell to divide and return to G1 phase, the MC must destabilize M_ss_ (black circle to white circle transition in Fig. 1A), causing Cdc20-bound APC/C (APC/C:Cdc20) to polyubiquitylate/degrade both securin and CycB (Peters, 2006), which allows sister chromatids to separate and the cell to proceed into A and T. As CycB-dependent kinase activity drops, the APC/C dissociates from Cdc20 and binds to Cdh1 (Hagting et al., 2002). The falling activity of APC/C:Cdc20 is the ‘spring’ that pulls the mitotic-exit gate closed and latched at the stable G1_ss_ state.

The irreversible ‘latching’ property of these gates guarantees that a proliferating cell alternates between S phase (DNA replication) and mitosis (accurate partitioning of replicated chromosomes to the two incipient daughter cells). A cell that leaves G1 and enters S phase is captured by the M_ss_ latch. The cell can only divide and return to G1 by destabilizing M_ss_ and getting captured by the G1_ss_ latch. The alternation between G1 and M is facilitated by ‘helper’ molecules (a starter kinase like CycE:Cdk2 and an exit protein like Cdc20). The helper molecules are regulated by negative feedback mechanisms that inactivate them after the transition is triggered (Novak and Tyson, 2022; Tyson and Novak, 2008). The latching behaviour requires that the control system alternate between two different steady states: G1_ss_ (low CDK activity and active CDK antagonists) and M_ss_ (high CDK activity and inactive CDK antagonists).

In this work, we show that this informal, verbal description of cell cycle progression is a precise mathematical consequence of the molecular interactions among the CDKs, antagonists and helpers of the mammalian cell cycle control system. Our mathematical model makes interesting predictions about the appearance of ‘endocycles’ (e.g. periodic DNA replication without mitosis, or periodic oscillations of CycB-dependent kinase activity without DNA replication) when the latching gates at G1 and M are compromised.

## RESULTS

### Proposed mechanism and mathematical model

At the heart of our model is APC/C:Cdh1, which is regulated by Cdh1-inhibitory phosphorylations by CycE-, CycA- and CycB-associated CDK activities (Fig. 1B; Peters, 2006). The double-negative interactions between Cdh1 and CycA- and CycB-dependent kinases are fundamental to the alternative stable steady states, G1_ss_ and M_ss_, of our model. Cdh1 is also regulated by early mitotic inhibitor 1 (Emi1; also known as FBXO5), which is an inhibitory substrate of APC/C:Cdh1 and accounts for a third double-negative feedback loop that renders APC/C:Cdh1 activity bistable (Cappell et al., 2018). To leave G1 phase and enter S/G2/M, APC/C:Cdh1 activity must be suppressed, and this is initiated by the transcription factor E2F. To prevent premature entry into S phase, E2F is inhibited in G1 phase by the retinoblastoma protein (Rb; also known as RB1), the primary agent arresting G1 cells at the restriction point. To pass RP, the cell must inactivate Rb by phosphorylation, started by CycD bound to Cdk4 and/or Cdk6 (the CDK activities are under the control of a variety of mitogens, growth factors and anti-growth factors). Rb phosphorylation releases E2F to induce the synthesis of CycE, CycA and Emi1. CycE and Emi1 combine to drive down Cdh1-dependent degradation of CycA and CycB. Both CycE and CycA can drive the cell into S phase, and phosphorylation of CycE targets it for ubiquitylation and degradation by the SCF pathway. CycA also activates the MuvB transcription factor complex for CycB expression (Fischer et al., 2022). Rising activities of CycA and CycB propel the cell through G2 into M phase. Cyclins E, A and B also amplify and prolong the phosphorylation of Rb started by CycD. During S phase, after the burst in E2F-mediated transcription, E2F is inactivated by phosphorylation by CycA and CycB-bound CDKs (Bertoli et al., 2013). Another kinase involved in APC/C:Cdh1 regulation is Polo (also known as PLK1). When Cdh1 activity is low (in S/G2/M), the accumulating Polo kinase is indirectly activated by CycA and CycB (Vigneron et al., 2018). Polo is responsible for phosphorylating Emi1, thereby promoting Emi1 degradation before mitotic entry (Hansen et al., 2004) and leaving A-type and B-type CDKs the only remaining activities to maintain Cdh1 inactive until mitotic exit.

The other central component of our model is CycB:Cdk1, whose activity drives the cell into mitosis and whose degradation allows the cell to exit mitosis (Nurse, 1990). CycB:Cdk1 complexes undergo inhibitory tyrosine-phosphorylation on the Cdk1 subunit by Wee1 and/or Myt1 kinases and dephosphorylation by Cdc25 phosphatases. The abrupt rise of Cdk1 activity at the onset of mitosis is triggered by the positive feedback loops between CycB:Cdk1 and Cdc25 and the double-negative feedback loop between CycB:Cdk1 and Wee1. [The bistability of this activation process (Novak and Tyson, 1993; Pomerening et al., 2003; Sha et al., 2003) creates the opportunity for an ‘unreplicated DNA’ checkpoint at the G2/M transition; an important check on genome integrity that we shall not pursue further in this paper]. Rising CycB:Cdk1 activity phosphorylates both APC/C and the kinase greatwall (Gwl; also known as MASTL). Phosphorylated APC/C (P-APC/C) rapidly binds to Cdc20, and the active complex (P-APC/C:Cdc20) promotes the degradation of both CycA and CycB. Meanwhile, activated Gwl phosphorylates and activates endosulfine (ENSA), which inhibits the major phosphatase [PP2A with the B55 subunit (PP2A:B55); B55 is also known as PPP2R2A] during mitosis (Gharbi-Ayachi et al., 2010; Mochida et al., 2010). As long as PP2A:B55 is inhibited, APC/C:Cdc20 actively clears CycA and CycB from the cell; however, as CycB:Cdk1 activity drops, the balance between APC/C phosphorylation and dephosphorylation shifts to favour its dissociation from Cdc20 and association with dephosphorylated Cdh1. These molecular changes drive the cell back to G1 (active APC/C:Cdh1). If Rb is active in the newborn cell, it will arrest at G1 (the RP).

Fig. 1B is hardly a complete picture of the complex web of molecular interactions governing progression through the mammalian cell cycle. Any ‘model’ of such molecular control systems must focus solely on those interactions that are essential to the issues under consideration. In this case, we are focusing on the interactions that create and control the ‘latching gates’ at the G1 and M steady states, and that generate the Cdh1- and Cdc20-driven endocycles observed when the gates fail to latch. To probe the properties of this model, we translate our proposed mechanism (Fig. 1B) into a set of ordinary differential equations (ODEs), in the Materials and Methods section, and we study the solutions of these ODEs by numerical simulation and by analytical methods based on bifurcation theory (Strogatz, 2014).

### A cell cycle clock

We start our analysis of the mathematical model by numerical integration of the ODEs in the absence of checkpoint regulation at RP or MC. With appropriate choice of kinetic parameters, numerical simulations exhibit persistent limit-cycle oscillations, corresponding to an autonomous cell cycle ‘clock’ (Fig. 1C). As expected, in G1 phase, Cdh1 is active and unphosphorylated Rb is high. As E2F activity rises, CycE is the first E2F target to appear, because it is not degraded by Cdh1. CycE phosphorylates Cdh1 and Rb, causing their activities to drop, allowing CycA and Emi1 to rise, which are hallmarks of the G1/S transition (Cappell et al., 2016, 2018). The rise of CycB is delayed until CycA activates the MuvB transcription factor complex. As CycB level rises, CycB:Cdk1 is activated by the positive feedback-aided dephosphorylation of Cdk1. High CycB-dependent kinase activity activates Polo and APC/C:Cdc20 and inactivates PP2A:B55 via the Gwl–ENSA pathway. Polo activation causes degradation of Emi1 (the Cdh1 inhibitor), but Cdh1-dependent APC/C activity remains low because high CDK activity phosphorylates Cdh1 and inhibits its association with APC/C. CycB-activated APC/C:Cdc20 maintains its activity until CycB is almost completely degraded, because the APC/C-inactivating phosphatase (PP2A:B55) is inhibited by ENSA.

### Mapping the cell cycle clock with bifurcation curves

The previous section illustrates that without any checkpoint control, our model of the mammalian cell cycle exhibits a limit cycle oscillation. To provide insight into this clock mechanism, we turn to bifurcation diagrams. A bifurcation diagram plots the steady state value of a cell cycle regulator as a function of increasing values of a bifurcation parameter. We choose CycE and Cdc20 as bifurcation parameters, because they act as helper molecules for the G1/S and the M/G1 transitions, respectively. To characterize the state of the cell cycle control system, we choose either Cdh1 activity or the level of CycB (mitotic cyclin). Given that the changes of the two helper molecules are almost out-of-phase during the cycle (see Fig. 1C), we set Cdc20=0 when calculating the bifurcation diagram for CycE, and CycE=0 for the Cdc20 diagram. To be more precise, to calculate the bifurcation diagram with CycE as the parameter, we eliminate the differential equations for both d[CycE]/d*t* and d[Cdc20]/d*t*, then set [Cdc20]=0 and [CycE]=constant everywhere in the remaining ODEs. We then solve for the steady state of the remaining nonlinear ODEs as a function of the value of [CycE], using the bifurcation software AUTO as implemented in XPP (Ermentrout, 2002).

**Fig. 2.**
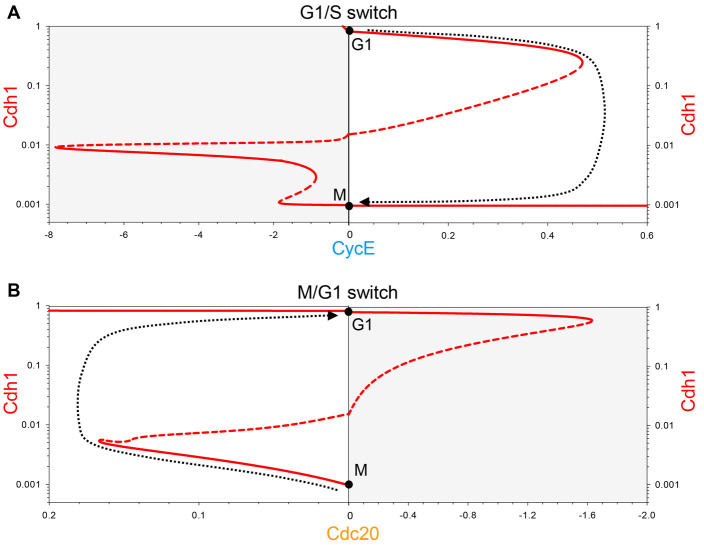
**Bifurcation diagrams for Cdh1 activity as a function of CycE or Cdc20.** (A) Steady state activity of APC/C:Cdh1 as a function of [CycE], and (B) as a function of [Cdc20]. Solid red curves: stable steady states; dashed red curves: unstable steady states; dotted black line: proposed cell cycle ‘trajectory’ projected onto the bifurcation diagram based on the negative feedback loops controlling CycE and Cdc20. To calculate the CycE diagram, we set [Cdc20]=0; for the Cdc20 diagram, we set [CycE]=0. The G1 and M steady states are marked by black circles. Notice that, for the CycE diagram, positive values of [CycE] are plotted to the right and negative values (shaded, which cannot be visited by the system) to the left. For the Cdc20 diagram, the positive and negative values are reversed so that the unshaded portions in panels A and B can be combined into a single panel in Fig. 3.

The Cdh1 bifurcation diagrams (Fig. 2) show a Z-shaped dependence of Cdh1 steady-state activity (the red curves) on the activity of each of the helper molecules, CycE (Fig. 2A) and Cdc20 (Fig. 2B). In Fig. 2A, we plot Cdh1 steady-state values for both positive values of CycE (white region to the right) and negative values (grey region to the left). (Although the negative region is ‘unreachable,’ its significance will become apparent later). Focusing on the white region, we see that, for 0<[CycE]<0.47, there are two coexisting, stable steady states of Cdh1 activity (on the upper and lower branches of the Z-shaped curve) separated by an intermediate branch of unstable steady states. The upper states are G1-like, and the lower states are S/G2/M-like. At [CycE]=0.47, the upper and intermediate branches merge and annihilate each other, leaving only a stable steady state of low Cdh1 activity. [CycE]=0.47, called a ‘saddle-node’ bifurcation point (Strogatz, 2014), represents the onset of the G1/S transition. Fig. 2B tells a similar story. For 0<[Cdc20]<0.17, there are two coexisting, stable steady states – an M-like state (high CycB activity) and a G1-like state (low CycB activity), separated by an intermediate branch of unstable steady states. At [Cdc20]=0.17, the M-like state is annihilated at a saddle-node bifurcation point, and, for [Cdc20]>0.17, the control system must leave the M state and switch to the branch of stable, G1-like steady states.

**Fig. 3.**
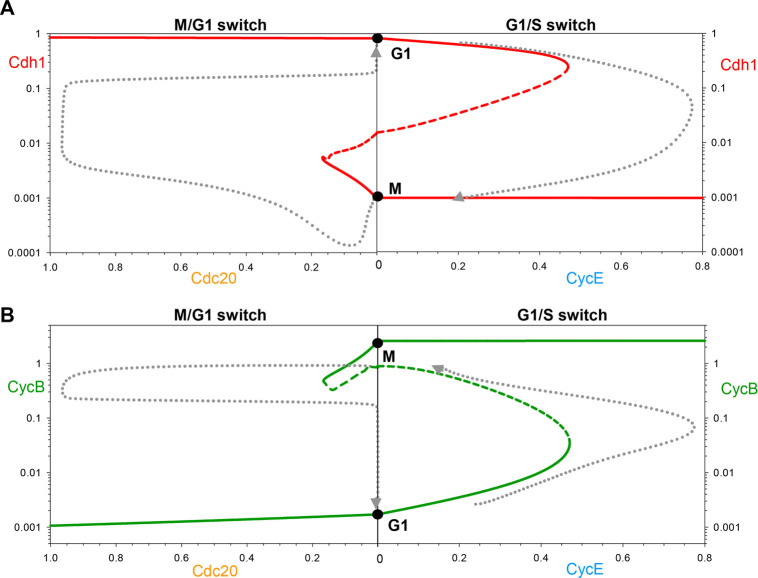
**Two complementary views of progression through the mammalian cell cycle.** (A) The changing activity of Cdh1 during progression through the mammalian cell cycle (Fig. 1C) is projected (grey dotted curve) onto a bifurcation diagram composed from the two panels in Fig. 2 (solid red curves: stable steady states; dashed red curves: unstable steady states). The negative feedback controls on CycE and Cdc20 are evident from the simulation, although they differ considerably from the proposed trajectories in Fig. 2. (B) CycB activity changes during the mammalian cell cycle projected onto a bifurcation diagram composed from the two panels in Fig. S1, with the same conventions as in panel A.

As long as the reverse transitions in both Fig. 2A and B occur for negative values of the switching variables, CycE and Cdc20, the G1/S and M/G1 transitions are irreversible. For instance, to leave G1 and enter S/G2/M, CycE activity must increase above 0.47 to get beyond the saddle-node bifurcation point (Fig. 2A). Thereafter, the trajectory drops to the branch of lower steady states, and, as CycE is degraded (as a consequence of CycE- and CycA-dependent phosphorylation and SCF-dependent ubiquitylation), the trajectory stops at M because it can go no further. To switch back to G1 phase spontaneously, [CycE] would have fall to negative values. For this reason, spontaneous ‘endocycles’ are impossible, and progression through the cell cycle is an irreversible alternation between G1/S and M/G1 transitions, as suggested by the cell cycle trajectory (dotted black curves in Fig. 2). However, any genetic or physiological disturbances that move the ‘unreachable’ saddle-node bifurcation points from negative to positive values of [CycE] or [Cdc20] could potentially create endocycles (G1/S/G1/S/… or M/G1/M/G1/…, respectively).

The corresponding CycB bifurcation diagrams (Fig. S1) are S-shaped, mirroring the Cdh1 curve (Fig. 2), because Cdh1 activity and CycB levels mirror each other. When [CycE] exceeds 0.47 (Fig. S1A), Cdh1 becomes inactivated and CycB level increases. Given that CycE is regulated by a negative feedback loop, its level decreases after the G1/S transition, as CycB is accumulating. As CycE level falls, Cdh1 does not become reactivated, because the reactivation threshold is at a negative value of [CycE]. Both CycA (not shown) and CycB reach stable steady-state values (M) as [CycE] tends to zero.

To reactivate Cdh1, the other helper molecule, APC/C:Cdc20, must be activated above a threshold value of 0.17 (Fig. 2B), which leads to the degradation of both CycA and CycB (Fig. S1B). Because APC/C:Cdc20 activity depends upon APC/C phosphorylation by CycB:Cdk1, Cdc20 activity falls as CycB activity falls (with a slight time delay). Cdh1, on the other hand, stays active and keeps CycB at a low steady-state level (G1) after Cdc20 inactivation. CycB does not spontaneously reaccumulate, because the CycB reactivation threshold is at negative Cdc20 value (−1.6). In this way, active Cdh1 latches the gate after the cell exits mitosis.

The dotted black trajectories in Fig. 2 and Fig. S1 are ‘sketched’ onto the bifurcation diagrams, assuming that Cdh1 and CycB activities change very rapidly relative to the rates of change of CycE and Cdc20, respectively. Indeed, that is the case for the parameter values used to compute Fig. 1C, where the transitions are very abrupt (the limit cycle has the characteristics of a ‘relaxation oscillator’). However, this assumption is not necessary – the transitions could be smoother without jeopardizing the ‘latching’ properties of the G1/S and M/G1 transitions. These properties depend solely on (1) the bistability of the control system, (2) the saddle-node bifurcations as the helper molecule activities rise, (3) the negative feedback loops that drive back down the helper molecule activities beyond the bifurcation point, and (4) the fact that the other saddle-node bifurcation associated with the Z- or S-shaped curves lies in the unreachable region of negative helper-molecule activities.

In summary, we propose that both G1/S and M/G1 transitions in the mammalian cell cycle are governed by irreversible bistable switches (‘latching gates’). To put together a picture of the whole cell cycle, we combine the two half-bifurcation diagrams calculated with CycE and Cdc20 as helper molecules (Fig. 3). Keep in mind that these diagrams are approximations based on our reasonable simplifying assumption that the two helper molecules do not coexist, that is that Cdc20 and CycE are absent (equal to zero) on the right and left sides, respectively. The combined Cdh1 bifurcation diagram maintains the characteristic Z-shape of the Cdh1 versus CycE and Cdh1 versus Cdc20 diagrams (Fig. 2A). Similarly, the combined CycB bifurcation diagram (Fig. 3B) maintains the S-shape of the diagrams in Fig. S1. According to our model, opening the G1/S gate triggers the transition from G1 to the alternative M steady state and also latches the M/G1 gate by inactivating Cdh1. To open the M/G1 gate, Cdc20 must be activated (in response to successful alignment of all replicated chromosomes on the metaphase spindle); during the transition from M to G1, Cdh1 is reactivated and the M/G1 gate is locked by degrading CycB. Alternation of the two switches is guaranteed by the licensing mechanism provided by the antagonism between CycB and Cdh1. The trajectory (grey dotted line) superimposed on Fig. 3 is derived from the numerical simulations of the model displayed in Fig. 1C. Fig. 3 confirms that the cartoon in Fig. 1A is indeed a precise consequence of the molecular mechanism in Fig. 1B, given reasonable assumptions on the rate laws and rate constants involved in the mathematical model.

To provide further evidence for our model, we next discuss mutations that interfere with the alternation of the two switches.

### Endoreplication cycles – Cdh1 endocycles

Mammalian cells, under certain conditions, exhibit endoreplication cycles, during which the cell undergoes multiple rounds of DNA replication without mitosis and cell division. (Under other conditions, a cell might exhibit over-replication, that is, persistent DNA synthesis exhibiting a steady rise in DNA content). In our view of cell cycle regulation, an endoreplicating cell does not visit the left sides of the diagrams in Fig. 3, rather it resets from G2 phase back to G1. Endoreplication can be induced in fission yeast cells by repressing synthesis of Cdc13, a B-type mitotic cyclin (Hayles et al., 1994), and in budding yeast cells by deleting five B-type cyclins (four mitotic and one S-phase cyclin; Haase et al., 2001). In fruit flies, both CycA and CycB are suppressed during endoreplication, which is driven by oscillating CycE:Cdk activity (Edgar and Orr-Weaver, 2001). In human cells, conditional inactivation (Itzhaki et al., 1997) or chemical inhibition (Gravells et al., 2013; Ma et al., 2009) of Cdk1 induces discrete rounds of DNA replication without mitosis or cell division. In these endoreplicating mammalian cells, Cdh1 activity is oscillating (Laronne et al., 2003; Ma et al., 2009) in the absence of any Cdc20 activation; the CycB level is also oscillating, although CycB:Cdk1 activity is suppressed. Therefore, we classify endoreplication cycles as Cdh1 endocycles.

These observations are consistent with the implications of our model that the irreversible nature of the G1/S switch (under normal cell cycling) requires CycB-dependent mitotic kinase activity. To illustrate this point, we have calculated the Cdh1 bifurcation diagram of the G1/S switch at different levels of Cdk1 inhibition (Fig. S2). The stronger Cdk1 inhibition is, the larger the Cdh1 reactivation threshold becomes. Above a critical value of Cdk1 inhibition (∼25% remaining Cdk1 activity), Cdh1 can reactivate at low CycE activity, rather than relying on Cdc20 activation. Hence, Cdh1 is still bistable at low Cdk1 activity (even at 0), but the G1/S switch loses its irreversible characteristic. At, say, 20% remaining Cdk1 activity, Cdh1 activity can oscillate with large amplitude as CycE activity oscillates back and forth across the two saddle-node bifurcation points (the C and Ↄ ‘noses’ of the Z-shaped bifurcation curve).

**Fig. 4.**
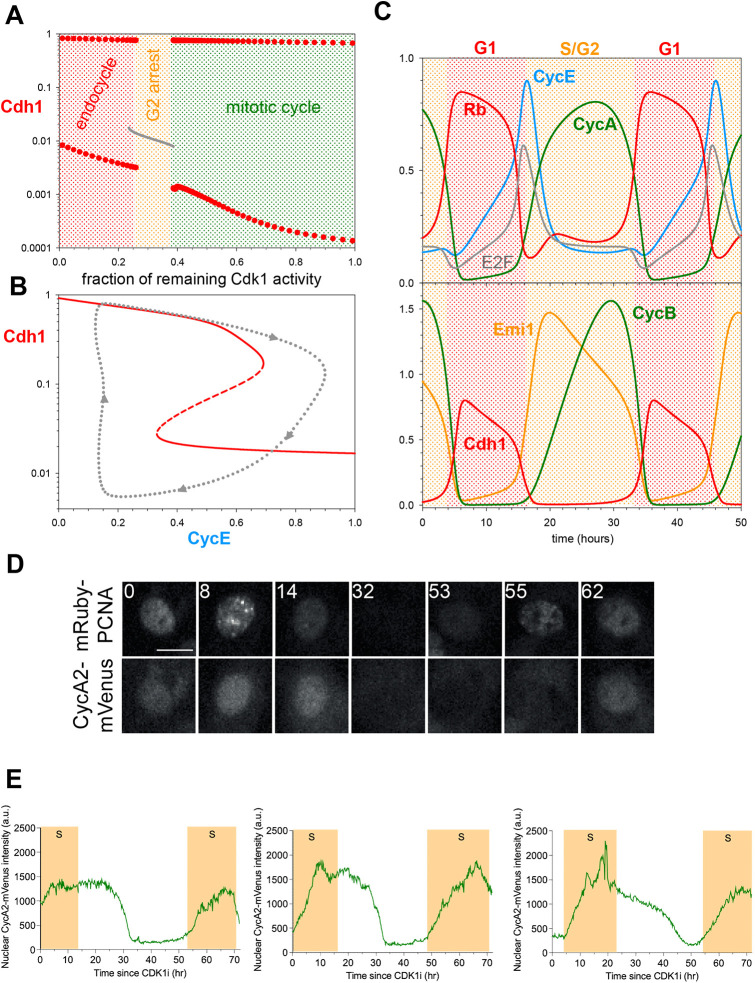
**Cdk1 inhibition converts mitotic cycles into Cdh1 endocycles.** (A) Bifurcation diagram: Cdh1 activity as a function of Cdk1 activity after chemical inhibition. Grey line: stable steady states; solid red circles: maximum and minimum excursions of Cdh1 activity during stable limit cycle oscillations. Mitotic cycles are distinguished from endoreplication cycles by the very low activity of Cdh1 (corresponding to high CycB:Cdk1 activity in mitosis). (B) Bifurcation diagram: Cdh1 activity versus CycE, for 10% remaining Cdk1 activity. As before, solid and dashed red curves indicate stable and unstable steady states, respectively; and the dotted grey line is the projection of Cdh1 limit cycle oscillations around a hysteresis loop on the bifurcation diagram. (C) Simulation of Cdh1 endocycles for 10% remaining Cdk1 activity. (D) Still images of mRuby–PCNA and CycA2–mVenus-labelled nuclei from timelapse experiments. Time shown in hours. Scale bar: 10 µm. (E) Graphs showing quantification of CycA2–mVenus in individual cells undergoing endocycles, plotted from the time of CDK1i addition (*t*=0 h). Shaded yellow areas represent S-phase, defined by mRuby-PCNA foci. *n*=1 with four technical replicates. a.u., arbitrary units.

Fig. 4 provides a closer view of how normal mitotic cycles are converted into Cdh1 endocycles (endoreplication cycles) as Cdk1 activity is suppressed by chemical inhibition. Mitotic cycles persist down to ∼60% inhibition of CycB:Cdk1 (Fig. 4A), with the only effect to extend the duration of G2 phase (not shown). For 26–38% of remaining Cdk1 activity, our model predicts a G2 block, because Cdk1 is unable to self-activate through the Wee1- and Cdc25-positive feedback loops. During this G2 arrest Cdh1 is kept inactive by combined inhibition from Emi1, CycA- and CycB-bound kinases. At above 75% inhibition of Cdk1 activity, Cdh1 cannot be kept inactive, but rather Cdh1 executes large amplitude oscillations around a hysteresis loop involving the bistable G1/S switch only (Fig. 4B). The trajectory on the Cdh1–CycE bifurcation diagram is a projection of the simulation shown on Fig. 4C. During this limit cycle oscillation, the periodic appearance of CycE and CycA induces initiation of DNA replication, and the concomitant inactivation of Cdh1 could lead to the accumulation of the replication licensing inhibitor, geminin (not present in our model). Subsequent degradation of Emi1 reactivates Cdh1 and resets the endoreplicating cell back to G1, when replication origins can be relicensed for a new round of DNA replication. Therefore, we expect the large amplitude Cdh1 oscillations to drive discrete rounds of DNA replication characteristic of endoreplicating cells.

To experimentally test our theoretical results, we first looked for endoreplication in non-transformed hTert-RPE1 (RPE1) cells after Cdk1 inhibition with the chemical inhibitor RO-3306 (Cdk1i). After 72 h treatment with Cdk1i, we observed distinct 8n and 16n peaks by flow cytometry, indicative of endoreplication (Fig. S3A,B). At high concentrations of Cdk1i (>7.5 µM) an increasing fraction of cells arrested in G1 (2n), presumably due to inhibition of Cdk2 at high concentrations of RO-3306, as previously reported (Ma et al., 2009). In timelapse imaging using the mRuby–PCNA reporter to track DNA replication (Zerjatke et al., 2017), we observed that endoreplication was even more prominent in 7.5 µM Cdk1i after depleting p53 from RPE1 cells using siRNA (Fig. S3C). Therefore, all subsequent experiments were performed under conditions of p53 depletion. To observe cell cycle dynamics in cells undergoing endocycles, we used timelapse imaging to quantify the levels of fluorescently tagged CycA2–mVenus in RPE1 cells (Mansfeld et al., 2011) co-expressing mRuby–PCNA during treatment with Cdk1i. In the absence of Cdk1i, CycA–mVenus showed characteristic oscillations for mitotic cycles, peaking in intensity during cell rounding (mitotic entry) followed by abrupt degradation (Fig. S3D). In cells treated with Cdk1i, an extended G2 was observed with initially high CycA2–mVenus levels that then dropped abruptly (Fig. 4D,E; Fig. S3F). In 60% of these cells, this extended G2 was followed by a new round of DNA replication in the absence of any signs of mitosis (endoreplication, Fig. 4D,E; Fig. S3B, Movies 1 and 2). These data support our theoretical predictions.

Another way to subvert the latching gate at M is by suppressing Emi1 synthesis, as suggested by experiments (Barr et al., 2016; Di Fiore and Pines, 2007; Machida and Dutta, 2007). According to our model, cells maintain their mitotic cycles up to ∼40% reduction of Emi1 synthesis (Fig. S4A). Stronger inhibition of Emi1 synthesis leads to an abrupt reduction in the amplitude of Cdh1 and Cdk1 oscillations (Fig. S4A,B). For nearly complete inhibition of Emi1 synthesis, the G1/S switch stops oscillating and settles onto a stable steady state. This steady state is characterized by intermediate values of Cdh1 and CycA activities, in addition to high CycE levels. We associate the reduced amplitude Cdh1 endocycles (caused by increased trough) and the intermediate Cdh1 steady states with continuous DNA synthesis (over-replication phenotype – when licensing and firing of replication origins are not temporally separated), based on the residual Cdh1 activity, which could maintain low levels of geminin, thereby allowing replication origin licensing and firing to proceed simultaneously.

### Cdc20 endocycles

**Fig. 5.**
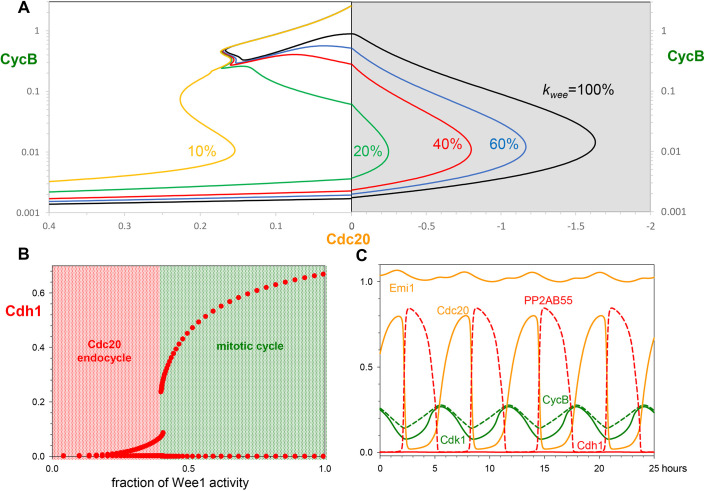
**Inhibition of Wee1 kinase activity converts mitotic cycles into Cdc20 endocycles.** (A) Bifurcation diagram: CycB activity as a function of Cdc20, for increasing inhibition of Wee1. (B) Bifurcation diagram: Cdh1 activity as a function of remaining Wee1 activity. (C) Simulated Cdc20 endocycles, for 10% Wee1 activity. The PP2AB55 trace is dashed to distinguish it from the Cdh1 trace (the solid red line at 0).

Given that Cdk1 inhibition disrupts the latching property of the M gate and enables Cdh1 endocycles, it is tempting to consider the consequences of the opposite effect: sustained CycB:Cdk1 activity. Working with HeLa cells, Pomerening et al. (2008) expressed an allele (*Cdk1AF*) for non-phosphorylatable Cdk1 subunits, which cannot be inactivated by Wee1 or Myt1 inhibitory kinases. Cdk1AF short-circuits the Cdk1 activation feedback loop operating at the G2/M transition (Fig. 1B). Cdk1AF-expressing cells carry out a relatively normal first mitosis, but then undergo rapid cycles of CycB accumulation and degradation at 3–6-h intervals. These fast CycB oscillations show certain resemblances to the early embryonic cell cycles of *Xenopus* (Dasso and Newport, 1990). Inspired by these experimental results, we decided to analyse the effects of weakening inhibitory Cdk1 phosphorylation in our model (Fig. 5). It is important to mention that the complete absence of Cdk1 inhibitory phosphorylation (Cdk1AF only) does not allow cell proliferation (Gupta et al., 2007) owing to premature entry into mitosis during S phase leading to mitotic catastrophe (Szmyd et al., 2019).

Fig. 5A presents CycB versus Cdc20 bifurcation diagrams for different values of *k*_wee_, the combined activities of Wee1 and Myt1 (hereafter, simply Wee1). Decreasing Wee1 activity moves the threshold for Cdc20 inactivation (the threshold for CycB re-accumulation) to less negative values of Cdc20 (i.e. to the left in Fig. 5A). When Wee1 activity falls below 13%, the Cdc20 threshold for CycB re-accumulation moves to positive values of Cdc20, meaning that exit from mitosis no longer latches the cell at the G1 gate. Now the control system can oscillate around a hysteresis loop on the CycB–Cdc20 bifurcation diagram. As the inhibitory phosphorylation of Cdk1 becomes weaker, the amplitude of the Cdh1 oscillations decreases (Fig. 5B) and finally becomes negligible at Wee1 activity of below 25%. In the absence of any fluctuations of Cdh1, the CycB level still shows persistent oscillations at low Wee1 activity (Fig. 5C). These oscillations of CycB level are exclusively driven by fluctuating activity of APC/C:Cdc20; so we call them Cdc20 endocycles. During Cdc20 endocycles, Cdh1 is kept inactive by high Emi1 levels and by strong inhibition by CycB:Cdk1 kinase (Fig. 5C). Given that the synthesis of both Cdh1 inhibitors is dependent on E2F activity (directly for Emi1 and indirectly – via CycA – for CycB), sustained Cdc20 endocycles require that the level of Rb must be less than the level of E2F. Indeed, these limit-cycle oscillations persist in the absence of Rb, providing an explanation for the observations by Pomerening et al. (2008) of Cdc20 endocycles in Rb-negative HeLa cells. We have experimentally tested for Cdc20 endocycles in Rb-positive RPE1 cells, which will be discussed after describing the role for Rb in the cell size checkpoint.

In summary, we have shown that inhibition and premature activation of the mitotic kinase has opposite effects on human cell cycle switches. Cdk1 inhibition breaks the latch at the M/G1 gate and induces Cdh1 endocycles, which trigger periodic and distinct rounds of DNA replications. In contrast, in the absence of inhibitory Cdk1 phosphorylation, the G1/S latch is broken, and CycB level oscillates rapidly by the periodic activation and inactivation of Cdc20.

### Checkpoints

Up to this point, we have been treating the cell cycle control network as an oscillator, which induces cell cycle events by measuring time only. However, this underlying clock is subject to several checkpoint mechanisms that make progression through the cell cycle sensitive to a variety of important intra- and extra-cellular signals. The most important signals are (1) extracellular growth and antigrowth factors, which govern passage through the restriction point, (2) cell growth, which must be sufficient to authorize the G1/S transition, (3) DNA damage, which can block both G1/S and the G2/M transitions, (4) unreplicated DNA, which blocks mitotic entry, and (5) misaligned chromosomes, which prevent the metaphase-to-anaphase transition. These checkpoint mechanisms stop progression around the cell cycle loop (Fig. 3) by creating stable steady states on the upper and lower branches of the bifurcation curves near the neutral point, where both CycE and Cdc20 are absent. In this subsection, we focus on two relevant checkpoints.

The mitotic checkpoint blocks activation of Cdc20 (thereby inhibiting degradation of CycB and securin) until all chromosomes become bioriented on the mitotic spindle (Musacchio, 2015). (Upon degradation of securin, active separase cleaves the cohesin rings that are holding sister chromatids together at bioriented centromeres, allowing the sister chromatids to be separated in anaphase). In the model, a reduction of Cdc20 activity to below ∼10% normal (not shown) terminates the limit cycle oscillation of CycB and creates a stable steady state of high CycB:Cdk1 activity.

**Fig. 6.**
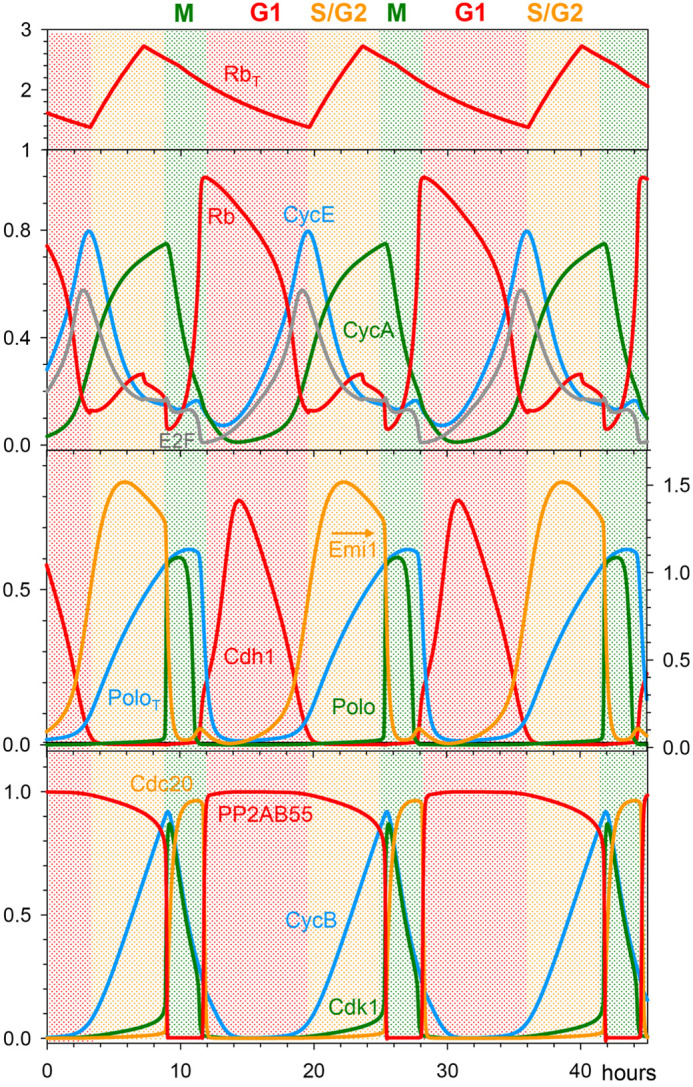
**Growth-controlled cell cycle upon Rb dilution.** The limit-cycle model is supplemented with cell cycle-regulated transcriptional control over Rb synthesis. Rb synthesis during S/G2 phase results in an increase of its concentration, followed during the remainder of the cell cycle by decreasing Rb concentration due to dilution by cell volume growth. (Notice that Rb concentration does not change during cell division). This mechanism automatically leads to two-fold fluctuations in Rb concentration when cell volume doubles over the course of a cell cycle. In the third panel, the arrow indicates that the variable is read off the right axis.

The effects of cell growth on cell cycle progression are complex and as yet not fully understood. However, it has been demonstrated that Rb plays an important role in size control (Zatulovskiy et al., 2020). At above a certain threshold concentration, Rb inhibits the G1/S transition by blocking E2F-dependent expression of CycE, CycA and Emi1. Our model is consistent with this observation because, at high Rb concentration, large amplitude mitotic oscillations of CycB become stabilized at a low, steady state concentration, which is characteristic of G1 phase (Fig. S5A). To illustrate the role of Rb in cell size control, we have supplemented our clock mechanism with an Rb-dilution model (Zatulovskiy et al., 2020). We assume that cells are growing linearly in volume and that the Rb synthesis rate is size-independent (proportional to the genome content) and transcriptionally regulated. Fast Rb synthesis is restricted to a 4-h-long window starting around the G1/S transition and leading to a doubling of Rb concentration; subsequently, Rb concentration is diluted out by volume growth during the remainder of the cycle (Fig. 6, top panel). Our assumptions provide a temporal pattern for cell cycle changes in the amount of Rb molecules (Fig. S5B) that agrees well with the experimental data of Zatulovskiy et al. (2020). In this framework, Rb concentration (amount/volume) mirrors the cellular DNA/volume ratio and provides a possible mechanism for balanced growth and division, by adjusting the period of the cell cycle to the time required to double cell mass (see Fig. S5A).

### Rb-controlled Cdc20 endocycles

**Fig. 7.**
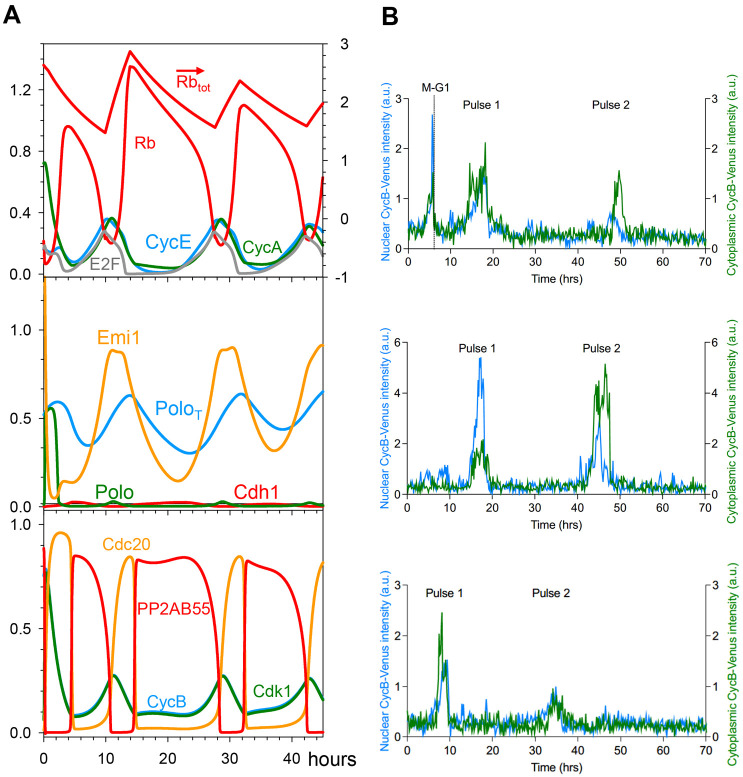
**Cdc20 endocycles controlled by Rb dilution.** (A) Numerical simulation of the growth-controlled cell cycle model with complete Wee1 inhibition (*k*_wee_=0). After exiting mitosis, CycB level shows small amplitude oscillations driven by APC/C:Cdc20 in the absence of any Cdh1 activity. Given that CycB:Cdk1 activity does not reach the mitotic threshold, both nuclear and cell division are hampered. The continuous rise in cell volume (not shown) causes an imbalance between Rb synthesis and dilution, which results in a decreasing amplitude of oscillations in Rb concentration. In the top panel, the arrow indicates that Rb_tot_ is read off the right axis. (B) Normalised CycB1–mVenus intensity in individual cells treated with Wee1 siRNA and undergoing Cdc20 endocycles. Blue curve: nuclear CycB level in arbitrary units (a.u.); green curve: cytoplasmic CycB level, in arbitrary units. Experiment shown is *n*=1 and is representative of three biological repeats.

We have tested the possibility that constitutively active CycB:Cdk1 could induce Cdc20 endocycles in the context of size control by an Rb-dilution mechanism. Our model predicts that inactivation of Wee1 after completion of mitosis induces small amplitude oscillations in CycB level, while Cdh1 is completely inhibited (Fig. 7A). Moreover, these Cdc20 endocycles have a period very close to the normal cycle time, because they are controlled by periodic synthesis and dilution of Rb in the following way. Cdc20 endocycles are driven by the fundamental negative feedback loop between CycB and Cdc20 (CycB:Cdk1 activates APC/C:Cdc20 and APC/C:Cdc20 degrades CycB). Given that CycB synthesis is initiated by CycA-dependent kinase and CycA is synthesized by E2F transcription factor in an Rb-dependent manner, Cdc20 endocycles (in Rb-positive cells) are controlled in part by the oscillating level of unphosphorylated Rb. Whenever unphosphorylated Rb is in stoichiometric excess over E2F the synthesis of both CycA and CycB are on hold and the oscillation is temporarily stopped.

We have used siRNA to deplete Wee1 inhibitory kinase in order to induce constitutively active CycB:Cdk1 complexes in Rb-positive RPE1 cells. In RPE1 cells with fluorescently tagged CycB1–mVenus (Collin et al., 2013), we used timelapse imaging to quantify CycB1 protein levels after Wee1 depletion. In control-depleted cells, CycB1–mVenus oscillates – increasing prior to mitotic entry (defined by cell rounding) and being rapidly degraded upon mitotic exit (Fig. S6A). After Wee1 depletion by siRNA (Fig. S6B), cells could go through an initial early mitosis but then continue to grow in volume, becoming large, interphase-arrested cells. Despite their robust interphase arrest, we observed cells that displayed one or two bursts of CycB signal, both in the cytoplasm and in the nucleus (Fig. 7B; Fig. S6C,D, Movies 3 and 4). The rise in CycB level was not accompanied by nuclear division.

In order to show that the drop of CycB level at the end of CycB pulses is caused by APC/C:Cdc20-dependent degradation, we analysed the kinetics of CycB degradation in control- and Wee1-depleted cells by estimating the half-life (*t*½) of CycB1–mVenus and its specific rate of degradation (dlnCycB/dt=ln2/*t*½) during normal mitotic exit and in the falling phase of the CycB pulses. At mitotic exit in control cells, the half-life of CycB is ∼10 min (Fig. S6E), consistent with a previous report (Collin et al., 2013), and its value is independent of the preceding peak of CycB. In contrast, the half-life of CycB is significantly longer and more variable in Wee1-depleted cells (Fig. S6E), which is a consequence of its hyperbolic (saturating) dependence on the CycB peak value (Fig. S6F). The peak value of CycB is a proxy for the maximum Cdk1 activity that is responsible for activating APC/C:Cdc20 in the pulses (Kraft et al., 2003), and the different kinetics of CycB degradation in control- and Wee1-depleted cells is a consequence of the elimination of the abrupt activation of Cdk1 in cells depleted of Wee1. Notice, however, that, in cells treated with siRNA against Wee1, the kinetics of CycB degradation are quite similar in both M/G1 peaks (normal exit from mitosis) and in CycB pulses, suggesting that CycB degradation in the pulses, like that in normal exit from mitosis, is APC/C:Cdc20 dependent (Kraft et al., 2003). That the activation of APC/C:Cdc20 in CycB pulses is mitosis-independent is supported by the observation of a lower Cdk1AF threshold for CycB degradation than for nuclear envelope breakdown (NEBD; Gavet and Pines, 2010).

We observed a similar, but less frequent, phenotype when we co-depleted Wee1 and Myt1 or inhibited Wee1 kinase activity using the small molecule inhibitor MK1775 (Fig. S7A–C). The majority of these cells arrested in mitosis, which is consistent with previous observations that a complete lack of inhibitory phosphorylation is not compatible with cell proliferation (Gupta et al., 2007; Szmyd et al., 2019).

In summary, our results support and extend the findings of Pomerening et al. (2008), who first described small amplitude CycB oscillations by weakening the Cdk1 inhibitory phosphorylation in HeLa cells. In Rb-negative HeLa cells, Cdc20 endocycles behave as an autonomous oscillator (Pomerening et al., 2008), whereas in Rb-positive RPE1 cells, the period of Cdc20 oscillations is influenced by an Rb-mediated size control mechanism (the present work).

## DISCUSSION

We have previously proposed that G1 and M are two alternative stable steady states of the budding yeast cell cycle control system (Chen et al., 2000; Novak and Tyson, 2022; Tyson and Novak, 2008). These alternative steady states are a consequence of double-negative feedback between B-type (Clb1–Clb5) CDKs (B-CDKs) and their antagonists (APC/C:Cdh1 and Sic1, a stoichiometric CDK inhibitor). Our toggle switch concept of the yeast cell cycle has been verified by elegant experiments in budding yeast (Cross et al., 2002; López-Avilés et al., 2009). Recently, we have shown that the toggle switch model also provides a natural explanation for two sorts of endocycles induced by perturbations of mitotic cyclin expression (Novak and Tyson, 2022): (1) endoreplication, where discrete rounds of DNA replication are induced by deletion of Clb1–Clb4 (the mitotic cyclins) and of Clb5 (one of the S phase cyclins) (Simmons Kovacs et al., 2012), and (2) Cdc14 endocycles, where periodic activation of the Cdc14 mitotic exit phosphatase occurs in the presence of a non-degradable form of the mitotic cyclin Clb2 (Lu and Cross, 2010; Manzoni et al., 2010).

In yeast, Cdh1 activity oscillates during both endocycles, and it promotes the degradation of the Nrm1 transcription inhibitor and of polo kinase (Cdc5) during endoreplication and Cdc14 endocycles, respectively.

Here, we propose that the mammalian cell cycle control network also supports two sorts of endocycles by a similar toggle switch mechanism. To this end, we introduce a mathematical model of mammalian cell cycling based on a molecular network of intermediate complexity, aiming to explain the mechanistic basis of endocycling, while maintaining a level of faithfulness to the temporal profiles of regulator activities and to the roles of checkpoint mechanisms in governing progression through the mammalian cell cycle. The mutual antagonism between the protein degradation pathway initiated by APC/C:Cdh1 and its target proteins CycA, CycB and Emi1 suggests that our toggle switch concept, originally proposed for yeast cells, also applies to the mammalian cell cycle. Indeed, hysteresis in the regulation of APC/C:Cdh1 activity is supported by experiments with mammalian cells (Cappell et al., 2018).

Our hypothesis is illustrated schematically in Fig. 1A. The bistable toggle switch (between APC/C:Cdh1 and CycB:Cdk1) is flipped ‘on’ (high CycB:Cdk1 activity) by CycE:Cdk2 and flipped ‘off’ (high APC/C:Cdh1 activity) by APC/C:Cdc20. We find that inhibition of mitotic CycB:Cdk1 complex makes APC/C:Cdc20 dispensable for Cdh1 reactivation by disabling the ‘latching’ property of the mitotic steady state (M_ss_), and converting the ‘one-way’ toggle switch into an autonomous oscillator regulated only by the remaining antagonistic interactions between APC/C:Cdh1 and CycA:Cdk2 plus Emi1. In the absence of mitotic CDK activity, cells are driven around a Cdh1 hysteresis loop by negative feedback regulation of the CycE:Cdk activity. The oscillations in CycE and CycA levels and their CDK activities lead to discrete rounds of DNA synthesis, analogous to yeast endoreplication cycles. We have confirmed this by live-cell imaging of fluorescently tagged CycA in RPE1 cells exposed to the Cdk1 inhibitor RO3306.

To date, numerous models of the mammalian cell cycle have been put forward. Most of these models focus on specific cell cycle transitions, but the work of Gérard and Goldbeter (2009) is particularly relevant to our work because it provides a detailed model of all phases of the mammalian cell cycle and even notes the possibility of endoreplication (Cdh1 endocycles). Pomerening et al. (2008) correctly surmised that the rapid Cdc20 endocycles they observed rely on a simple negative feedback loop involving CycB, Cdk1 and Cdc20, and that these oscillations are normally overridden by a ‘bistable switch’ that toggles between interphase (low CycB:Cdk1 activity) and mitosis (high CycB:Cdk1 activity); but they did not back up this hypothesis with a mathematical model. To our knowledge, there are no mathematical models that account for both Cdh1 and Cdc20 endocycles in mammalian cells, or that provide a general dynamical theory of how these endocycles arise and how cells avoid their potentially deleterious consequences.

The current model can be compared to that of Tyson and Novak (2001), where the molecular mechanism regulating the transitions between G1 and S/G2/M phases was studied by mathematical modelling. The 2001 model focused on normal cycling (G1-S-G2-M) driven by cell growth, where the G1/S transition was controlled by a saddle-node bifurcation, but progression through S/G2/M and back to G1 was driven by an autonomous negative feedback loop (see Fig. 4 in the paper). Here, by supplementing the 2001 model with other crucial proteins and interactions, we show that the double-negative feedback loop that stabilises the G1 and M steady states is sufficiently strong to render both transitions (G1/S and M/G1) irreversible. We show that specific mutations of the feedback loops can modify the bistability range of one of the underlying switches (the G1/S or M/G1 ‘gate’), potentially making the transition reversible and thereby giving rise to endocycles.

This mechanism of endoreplication, suggested by our theoretical model and verified experimentally, provides a basis for understanding how whole-genome doubling (WGD) can arise during tumorigenesis. The many layers of regulation underlying our ‘latching’ mechanism for cell cycle progression ensure that WGD is a rare event. However, it is estimated that up to 40% of all cancers have undergone at least one WGD event (Bielski et al., 2018). WGD can promote tumorigenesis by buffering the effects of deleterious mutations, by fostering mutations that increase cell proliferation (Dewhurst et al., 2014; López et al., 2020; Quinton et al., 2021), and – quite generally – by disrupting the genomic stability of cells (Fujiwara et al., 2005). By providing a mechanistic basis for how WGD can arise, our model might assist efforts to develop targeted treatments against WGD.

Endoreplication, is often induced by mutations that short-circuit mitosis by reducing or eliminating CycB-dependent kinase activity. The inverse perturbation, inducing mitosis in the presence of non-degradable CycB, generates Cdc14 endocycles in yeast cells. In mammalian cells, persistent mitotic Cdk1 activity induced by non-degradable CycB reactivates the error-correction mechanism of the mitotic checkpoint, which results in sister chromatids oscillating between the two poles (pseudo-anaphase) (Vázquez-Novelle et al., 2014; Wolf et al., 2006). These oscillations are the consequence of tension-dependent fluctuations of Aurora B kinase activity at kinetochores. We have investigated an alternative way to disrupt the antagonistic relationship between mitotic kinase and APC/C:Cdh1 in mammalian cells, by depleting cells of Wee1 kinase, the kinase that inhibits CycB:Cdk1 activity in G2. We find that sustained activity of CycB:Cdk1 in Wee1-depleted cells makes the CycE:Cdk dispensable for Cdh1 inactivation, because it maintains constitutively phosphorylation of Cdh1 and hence keeps it inactive. Moreover, in the absence of inhibitory phosphorylation of CycB:Cdk1, APC/C:Cdc20 is activated prematurely, which promotes early degradation of CycB and (because of the negative feedback loop between CycB and Cdc20) loss of APC/C:Cdc20 activity. Hence, although CycB:Cdk1 activity is ‘sustained’ under these conditions, the amplitude of CycB:Cdk1 oscillations is never high enough to drive the cell into mitosis or low enough to let Cdh1 make a comeback. Therefore, sustained activity of CycB:Cdk1 induces Cdc20 endocycles in the absence of Cdh1 activity, which makes the situation in human cells different from Cdc14 endocycles in yeast where Cdh1 oscillates. This dissimilarity between yeast and human cells could be a consequence of different mitotic exit phosphatases and their regulation, as well as different roles of Cdc20 and Cdh1 in the degradation of mitotic CycBs. In budding yeast, complete degradation of the Clb2 mitotic cyclin requires Cdh1, which is dephosphorylated during mitotic progression by the release of the active Cdc14 phosphatase from the nucleolus (Bardin and Amon, 2001). In contrast, in human cells, Cdh1 is dispensable for degradation of mitotic cyclins, and their mitotic exit phosphatase, PP2A:B55, is kept inactive by CycB:Cdk1 via the Gwl–ENSA pathway. Despite these differences, notice that Cdc20 fluctuations induced by sustained CycB:Cdk1 activity are accompanied by large amplitude oscillations of PP2A:B55 phosphatase activity (Fig. 7A). This observation suggests that unregulated Cdk1 activity induces mitotic exit phosphatase endocycles in both yeast and human cells.

On the experimental front, we have demonstrated these small amplitude oscillations in CycB level using live-cell imaging of RPE1 cells depleted for Wee1 by siRNA. A kinetic analysis of interphase CycB pulses suggests that the pulses are APC/C:Cdc20 dependent, consistent with our model; however, the Cdc20 dependence of the pulses still awaits direct experimental proof. Unfortunately, we have been unable to achieve efficient or sustained inhibition of Cdc20 activity, either by siRNA or by use of the APC/C inhibitors (ProTAME and APCin). The periods of CycB oscillations that we observe in RPE1 cells are significantly longer than those for the CycB oscillations observed by Pomerening et al. (2008) in HeLa cells, which we attribute to the indirect role of an Rb-dependent size-control mechanism on the production of CycB. Importantly, Wee1 inhibitors are currently in clinical trials for cancer treatment (Gorecki et al., 2021). The aim of these inhibitors is to specifically target cancer cells on the basis that only p53-mutant cancers, which rely on Wee1 to maintain the DNA damage checkpoint in G2, will be sensitive to Wee1 inhibitors (Hirai et al., 2009; Otto and Sicinski, 2017). By providing an understanding of the effects of inhibiting Wee1 in non-cancerous cells, our model might allow for a better understanding of potential side-effects of this treatment.

We have simplified our human cell cycle model by neglecting some cell cycle regulators, including cyclin-dependent kinase inhibitors (CKIs) like p27 (CDKN1B), p21 (CDKN1A) etc. These CKIs provide an extra layer of antagonism to the regulatory network (CKIs inhibit CDKs and are targeted to degradation by CDKs). There is no theoretical bottleneck to extend our model with CKIs, and this is a task for future work. For instance, p27 has a complex role in regulating the activities of CycD-bound Cdk4 and Cdk6 and CycE:Cdk2 (Guiley et al., 2019), thereby influencing the G1/S transition by interfering with the Rb–E2F double-negative feedback loop. p21 plays similar roles in the DNA-damage response induced by p53 (Brugarolas et al., 1999; He et al., 2005).

## MATERIALS AND METHODS

### Computational methods

#### Cell cycle clock model

The mathematical model presented here describes the biochemical interactions governing the mammalian cell cycle control network. It is assumed that the activity of each cyclin:CDK heterodimer is limited by the availability of cyclin subunit, which strongly and rapidly binds to its CDK partner. In early G1, cyclin expression is repressed via Rb-dependent stoichiometric inhibition of E2F transcription factors. A fraction of total Rb (Rb_tot_) protein is mono-phosphorylated and inactivated by CycD bound to Cdk4 and Cdk6 (the CycD parameter here); the remaining fraction of unphosphorylated Rb (Rb_t_) is:

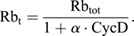
This pool of unphosphorylated Rb can be further phosphorylated by the other cyclin:CDK complexes, such that the rate law of Rb available to inhibit E2F (i.e. Rb molecules that are not phosphorylated by any cyclin:CDK complex) is given by the differential equation:

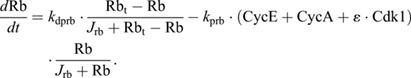
Notably, *ε* is a parameter that quantifies the relative activity of Cdk1. We set *ε*=1, unless it is reduced to a value 0<*ε*<1, to simulate Cdk1 inhibition, as indicated in the text. We are using Michaelis–Menten kinetics to describe the rates of phosphorylation (‘prb’) and dephosphorylation (‘dprb’) of Rb. Next, assuming that the Rb:E2F complex (RbEF2) is in equilibrium with the dissociated monomers, we calculate its concentration by:

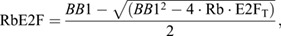
where *BB*1=Rb+E2F_T_+*K*_drbe2f_, E2F_T_ is the total concentration of E2F (assumed to be constant), and *K*_drbe2f_ is the equilibrium-dissociation constant of the complex. In addition, E2F can be independently inhibited through CDK-dependent phosphorylation:

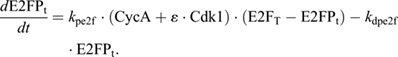
Consequently, the fraction of active E2F is given by:

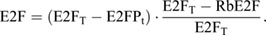
Active E2F (i.e. unbound by Rb and not phosphorylated by CDKs) stimulates the transcription of a number of genes required for G1/S progression, including CycE, CycA and Emi1.

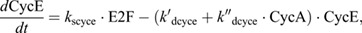


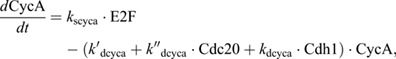


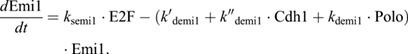


In addition to being regulated transcriptionally, these proteins are also targeted for degradation in specific manners, as described by the ‘*k*_d…_’ terms in these differential equations. CycA is a substrate of the ubiquitin ligase APC/C in complex with either Cdc20 or Cdh1; CycE is a substrate of the SCF ubiquitin ligase, after it is phosphorylated by CycA:Cdk2; and Emi1 is a target of both APC/C:Cdh1-mediated degradation and SCF-mediated degradation (after phosphorylation by Polo kinase). For these reasons, CycE – but not CycA or Emi1 – accumulates in G1; in S phase, CycE is rapidly degraded in response to CycA-mediated phosphorylation; and during M phase, both CycA and Emi1 are rapidly degraded (by different pathways) and kept low throughout G1. All of these regulators cooperate to drive the inactivation of Cdh1 at the G1/S transition. The stoichiometric binding of Emi1 to Cdh1 is modelled in the same way as the binding of Rb to E2F, namely:

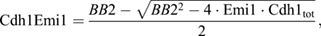


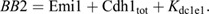


The phosphorylation of Cdh1 by CycE, CycA and CycB is described by:

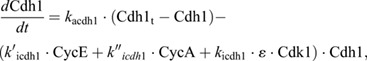
where Cdh1_t_ is the Emi1-free Cdh1: Cdh1_t_=Cdh1_tot_−Cdh1Emi1.

As CycA accumulates, it is responsible for driving the accumulation of CycB and Polo:

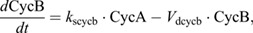


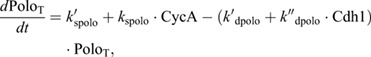
with *V*_dcycb_ being a degradation rate function that depends on Cdc20 and Cdh1:

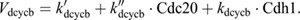
Nevertheless, as the CycB:Cdk1 complex accumulates, it is initially inactivated by Wee1-dependent phosphorylation; the active, dephosphorylated form is denoted as Cdk1:

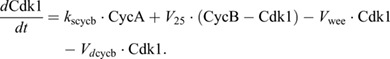
The net rate of accumulation of the dephosphorylated CycB:Cdk1 complex depends on the rate functions for the Wee1 kinase and Cdc25 phosphatase reactions:

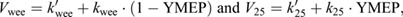
where YMEP is a Goldbeter–Koshland function for the tyrosine-modifying enzymes:

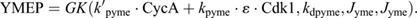
The GK function depends on the activities of CycA, Cdk1 and a constitutive phosphatase, denoted by the constant parameter *k*_dpyme_. The GK function is defined as:

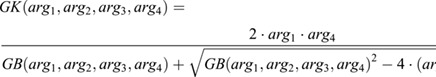
where

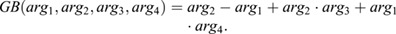


The GK function describes the steady-state ratio of phosphorylated-to-dephosphorylated substrate, which is a sigmoidal function of kinase activity when the kinase and phosphatase enzymes are operating near saturation (i.e. ‘zero-order’ ultrasensitivity). We use the GK function for mathematical convenience, even though the kinase and phosphatase enzymes are unlikely to be operating near saturation. A more likely basis for the ultrasensitive response is distributive multi-site phosphorylation of Wee1 and Cdc25 (Kim and Ferrell, 2007; Lu et al., 2012), but the GK function is easier to implement in a system of differential equations.

Together, CycA and Cdk1 also lead to the activation of Polo and Gwl kinases:

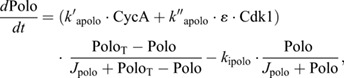


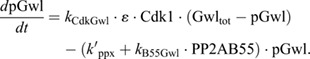
In addition, Gwl is dephosphorylated by the PP2A:B55 phosphatase. In its active, phosphorylated form, Gwl phosphorylates ENSA (pENSA_t_), which leads to the formation of an inhibitory complex with the PP2A:B55 phosphatase:

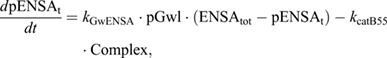
where Complex=B55_tot_−PP2AB55, B55_tot_ being the total concentration of B55, assumed to be constant. The dissociation of the complex is favoured by the PP2A:B55-dependent dephosphorylation of pENSA:

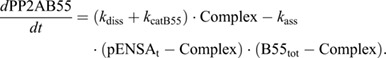
Finally, when the ratio of Cdk1 and PP2AB55 increases sufficiently, Cdc20 is activated, leading to the degradation of mitotic cyclins:

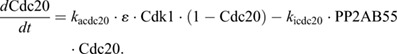


#### Size control model

The rate of cell volume growth is assumed to be constant (see Fig. 1K in Zatulovskiy et al., 2020):

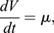
and volume is halved at cell division when Cdk1 drops below 0.7. In order to model size-controlled cycling, the total Rb concentration is converted from a constant into a dynamic variable, where the rate of synthesis in concentration units is inversely proportional to the volume:

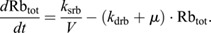
The rate of Rb synthesis (*k*_srb_) is assumed to change in a cell cycle-dependent manner. During G1, *k*_srb_ is very small (0.02 h^−1^), which means that the amount of total Rb protein is roughly constant, given a sufficiently long (∼30 h) half-life (*k*_drb_=0.023 h^−1^). Consequently, the protein concentration depends on the cellular volume at this stage, or in other words, the rate of change of Rb_tot_ concentration depends on the rate of volume growth, *µ*. Nevertheless, the amount of Rb must be replenished during each cycle; to this end, we assume that Rb expression is turned on (*k*_srb_=0.1 h^−1^) after S-phase entry (when CycA>0.3) for a fixed duration (4 h), ensuring that a fixed amount of protein is expressed during each cycle. This amount corresponds to a doubling of the Rb number of molecules present in early G1.

#### Computation

Solutions to the system of differential equations introduced above have been calculated numerically, using the XPPAUT software package with the ‘Stiff’ integration method. The XPPAUT code is provided in Table S2. The numerical values of the parameters are provided in Table S1, unless otherwise stated.

#### Bifurcation diagram calculation

Bifurcation diagrams of the system were calculated using the AUTO extension of XPP. Given our assumption that there is no significant activity overlap between the two helper molecules, CycE and Cdc20, the differential equations describing the two species were replaced by parameters with the same name. Thus, to plot the bifurcation diagrams with respect to CycE, Cdc20 was set to zero, and the steady state solutions of the system were calculated for a range of CycE values. Cdc20 bifurcation diagrams were calculated analogously.

### Experimental methods

#### Cell maintenance

hTert-RPE1 cells (ATC) were maintained in DMEM (Gibco) with 10% FBS (Gibco) and 1% penicillin-streptomycin (P/S; Gibco) at 37°C and 5% CO_2_. Cells were passaged every 3–4 days and tested for mycoplasma by ELISA every month. CyclinB1–mVenus RPE1 cells were provided by Jonathon Pines (Institute of Cancer Research, London, UK) and first described in Collin et al. (2013). CyclinA2–mVenus mTurquoise-H2B mRuby-PCNA cells were provided by Joerg Mansfeld (Institute of Cancer Research, London, UK) and first described in Mansfeld et al. (2011).

#### Cell cycle analysis by flow cytometry

hTert-RPE1 cells were seeded at 30% confluency into six-well tissue culture plates 1 day before treatment. The next day, DMSO (vehicle control) or different concentrations of the CDK1i RO-3306 (both Selleckchem) were added to wells and left in for 72 h. After 72 h, cells were washed 1× in PBS, trypsinised and centrifuged at 1000 ***g*** for 5 min at 4°C. The cell pellet was washed one more time in PBS, before cells were resuspended in 300 µl of PBS. Cells were then fixed by adding 700 µl of 100% ice-cold ethanol and at kept at −20°C overnight. The next day, cells were washed in ice-cold PBS and stained with 20 µg/ml PI solution  (Sigma) in PBS in 0.1% Triton X-100 and 200 µg/ml DNase-free RNaseA (Thermo Fisher Scientific) for 30 min, in the dark at room temperature. Stained cells were strained through 0.2 µm cell strainers into FACS tubes (BD) and analysed on the BD FACS Symphony analyser. Cell cycle analysis was performed in FlowJo.

#### Cyclin A2–mVenus timelapse experiments

hTert-RPE1 mTurquoise–H2B mRuby–PCNA Cyclin A2–Venus cells were reverse transfected in 384w PhenoPlates (PerkinElmer) with non-targeting control (NTC) or TP53 siRNA (ONTargetpools, Horizon Discovery). Cells were transfected with 20 nM siRNA using 40 nl/well of Lipofectamine RNAiMAX (Thermo Fisher Scientific) diluted in 10 µl OPTIMEM (Gibco). 1000 cells/well were plated on top of the transfection mix in 20 µl of phenol-red free DMEM (with 10% FBS and 1% P/S) and incubated for 24 h. Before imaging, cells were treated with either DMSO or 7.5 µM of the CDK1 inhibitor (CDK1i), RO-3306. A breathable membrane was applied over the plate and cells were imaged every 10 min on the Operetta CLS high-content microscope (PerkinElmer) at 37°C and 5% CO_2_ for 72 h using the 20× NA 0.8 objective. Background subtraction was performed in FIJI and cells were manually tracked to determine S-phase and quantify CycA2–mVenus intensity. Manual tracking was necessary to accurately quantify CycA2–mVenus levels in highly motile RPE1 cells over the 72 h time course.

#### Cyclin B1–mVenus timelapse experiments

hTert-RPE1 Cyclin B1–mVenus cells were reverse transfected in Ibitreat 8-well Ibidi chambers with NTC (non-targeting control), Wee1 or Wee1 and Myt1 siRNA (ONTargetpools, Horizon Discovery). Cells were transfected with 20 nM siRNA using 0.16 µl Lipofectamine RNAiMAX (FisherScientific) diluted in 40 µl/well OPTIMEM. 6000 cells/well were plated on top of the transfection mix in 300 µl of phenol-red free DMEM (with 10% FBS and 1% P/S) and left for 6 h. The Wee1 inhibitor, MK1775 (Selleckchem) was added to a final concentration of 2.5 µM immediately prior to imaging. Cells were imaged every 10 min on the inverted Olympus IX83 microscope with spinning disk unit at 37°C and 5% CO_2_ for 72 h using the 20× 0.7 NA objective. Background subtraction was performed in FIJI software and cells were manually tracked to quantify CycB1–mVenus nuclear intensity. Manual tracking was necessary to accurately quantify CycB1–mVenus levels in highly motile RPE1 cells over the 72 h time course.

#### Western blotting

hTert-RPE1 Cyclin B1–mVenus cells were reverse transfected in 24-well plates with 20 nM NTC or Wee1 siRNA using 1 μl of Lipofectamine RNAiMAX (Thermo Fisher Scientific) diluted in 100 µl/well OPTIMEM. Cells were plated on top of the transfection mix in 400 µl of DMEM (with 10% FBS and 1% P/S) and left for 6 h. Cells were then washed in 1× PBS lysed in 50 µl of 1× Laemmli buffer and cell lysates were loaded and run into 4–20% Tris-Glycine Novex pre-cast gels (Thermo Fisher Scientific). Separated proteins were transferred to PVDF-FL membranes which were then blocked in blocking buffer (5% milk in TBS with 10% glycerol) for 1 h at room temperature. Antibodies raised against Wee1 (CST 4936, 1:500), Myt1 (CST 4282, 1:1000), pY15-CDK (ab133463, 1:1000), β-actin (CST 3700, 1:1000) and vinculin (CST 13901, 1:2000) were diluted in blocking buffer and incubated with membranes overnight at 4°C. The next day, membranes were washed three times for 10 min each time in TBS with 0.05% Triton X-100 (TBS/T) and then incubated for 1 h at room temperature in anti-rabbit-IgG secondary antibody conjugated to HRP diluted 1:2000 in blocking buffer. Membranes were washed three times for 10 min each time in TBS/T and developed using Bio-Rad ECL substrate. Blots were imaged on an Amersham Imager 600. Uncropped western blots are shown in Fig. S8.

## Supplementary Material



10.1242/joces.261364_sup1 Supplementary information
