# Supplementary material for: The oscillation of mitotic kinase governs cell cycle latches in mammalian cells
Source: J Cell Sci. 2024 Feb 13;137(3):jcs261364. doi: 10.1242/jcs.261364 (PMC10911285; doi:10.1242/jcs.261364)
Supplement: Supplementary information [file joces-137-261364-s1.pdf]

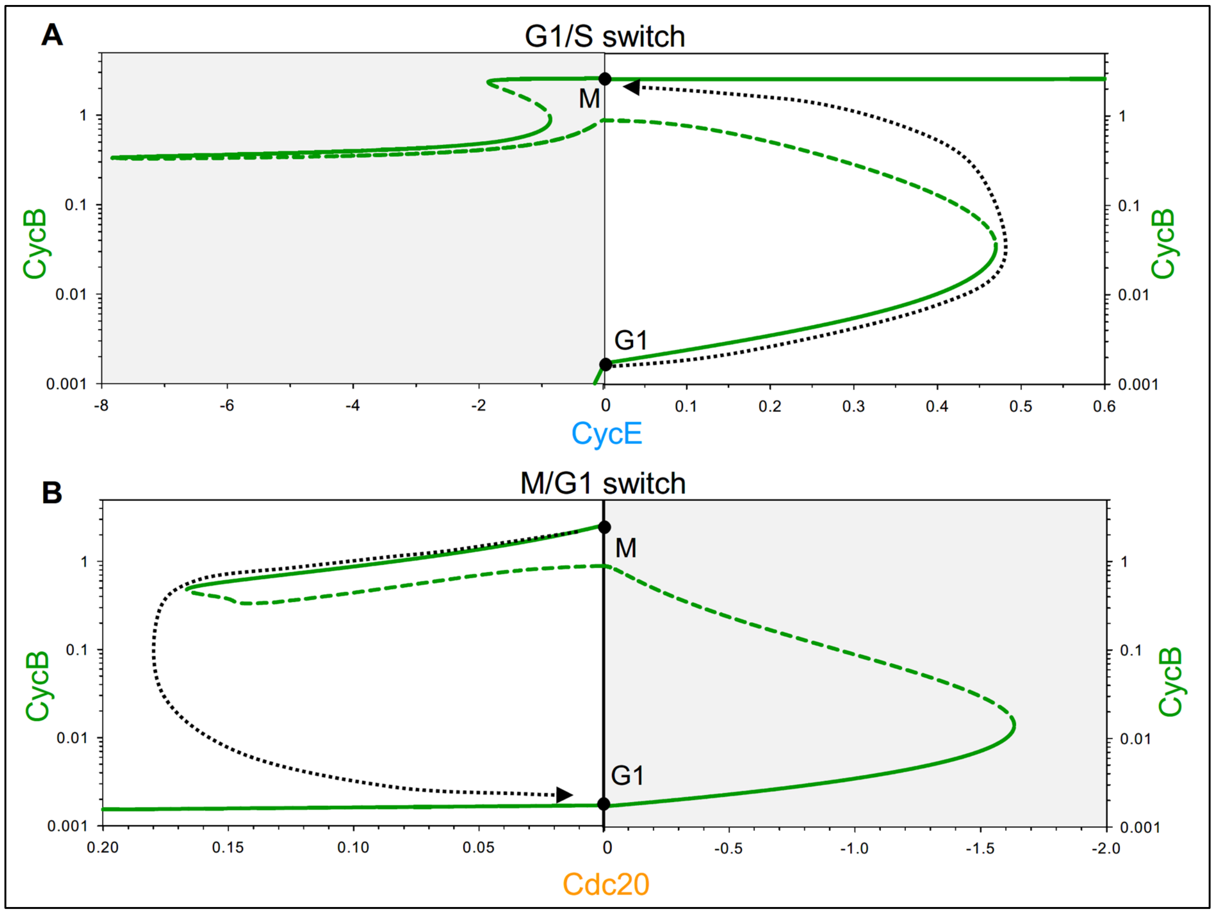

Fig. S1. Bifurcation diagrams for CycB level as a function of CycE or Cdc20. Related to Fig. 2. See details in Fig. 2.

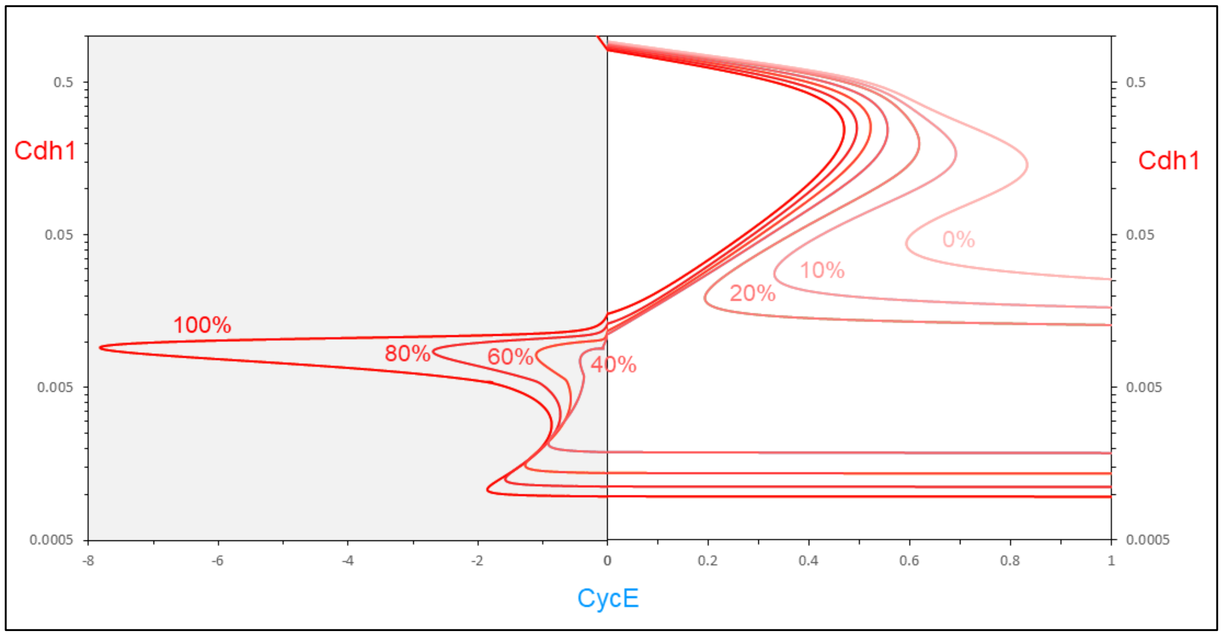

Fig. S2. Bifurcation diagrams that account for Cdh1 endocycles. Related to Fig. 4. Cdh1 vs CycE bifurcation curves for increasing inhibition of Cdk1 activity. Percentage refers to level of Cdk1 activity remaining, i.e., 100% means full activity, 0% is no activity. If Cdk1 activity is < 25%, the gate at **M** (CycE = 0, Cdh1 low) fails to latch. As CycE level drops, Cdh1 will spontaneously reactivate.

Journal of Cell Science • Supplementary information

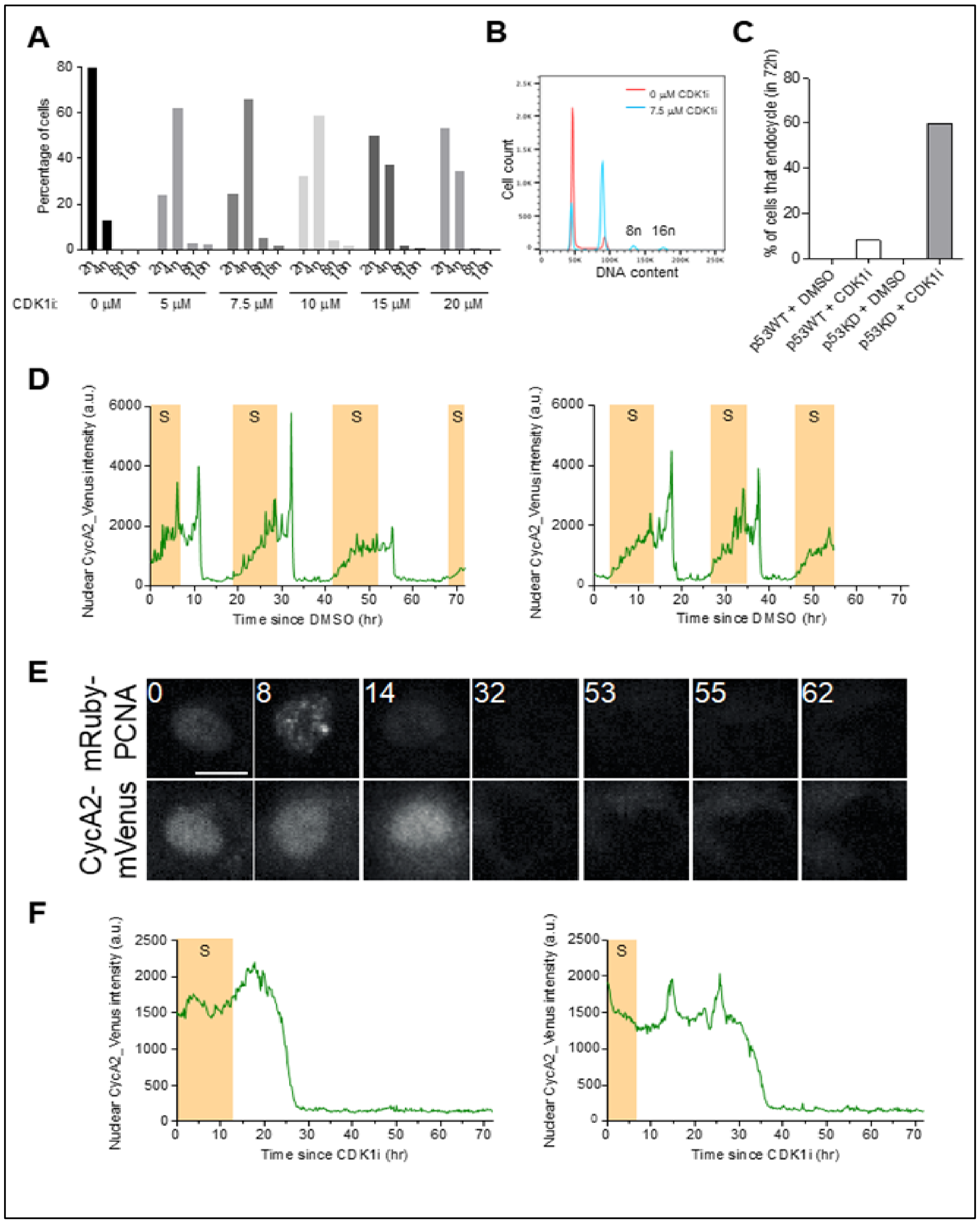

Fig. S3. Cdk1 inhibition-induced endoreplication (Cdh1 endocycles). Related to Fig. 4. (A) The percentage of cells of different ploidy, as quantified by flow cytometry, after treatment with different doses of the Cdk1i, RO-3306, for 72 h. Discrete 8n and 16n peaks are characteristic of endoreplication. **(B)** FACS plot of RPE1 cells treated with DMSO (0 μM CDK1i) or 7.5 μM of CDK1i for 72 h. Discrete 8n and 16n peaks are visible, indicative of endoreplication. For A and B, n=1 is shown, representative of two biological repeats. **(C)** The percentage of cells undergoing at least one endocycle in the 72 h imaging window in each condition. WT is wild-type p53. KD is p53 knockdown by siRNA. **(D)** Fluctuations of CycA2-mVenus during mitotic cycles in the presence of vehicle (DMSO). **(E)** CycA2-mVenus in individual cells that do not undergo endocycles. Still images of mRuby-PCNA and CycA2-mVenus labelled nuclei from timelapse experiments. Time shown in hours. Scale bar is 10 μm**. (F)** Graphs showing quantification of CycA2-mVenus, plotted from the time of CDK1i addition (t = 0 h). Shaded yellow areas represent S phase, as defined by mRuby-PCNA foci. n=1 with four technical repeats.

Journal of Cell Science • Supplementary information

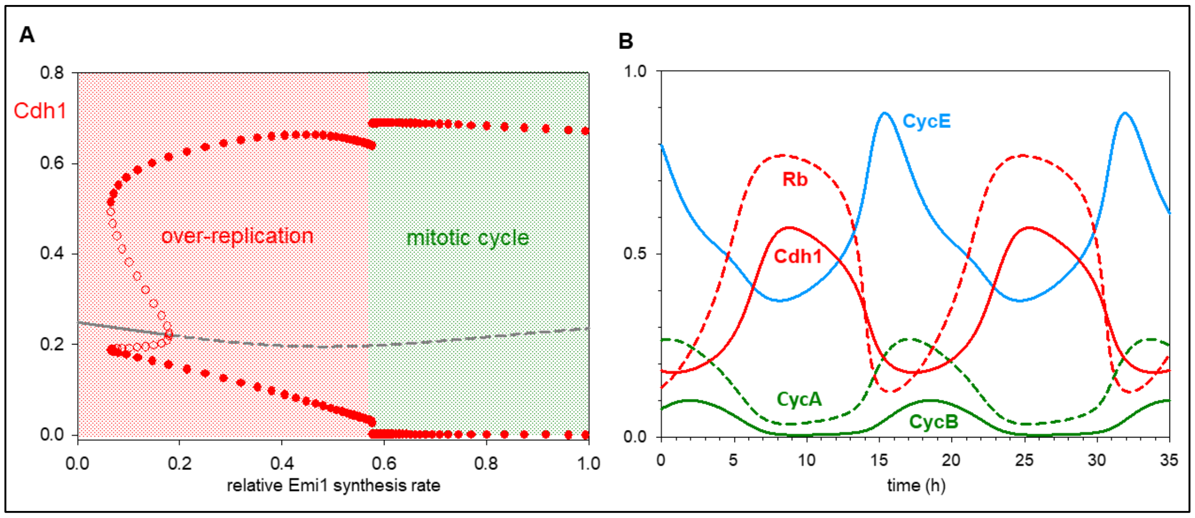

Fig. S4. Down-regulation of Emi1 synthesis converts mitotic cycles into over-replication cycles. Related to Fig. 4. (**A**) Bifurcation diagram: Cdh1 activity as a function of the relative synthesis rate of Emi1. Solid (dashed) gray line: stable (unstable) steady states; solid (open) red circles: maximum and minimum excursions of Cdh1 activity during stable (unstable) limit cycle oscillations. Notice that Cdh1 axis is linear compared to logarithmic on Fig. 4A. (**B**) Simulation of Cdh1 endocycles for 90% suppression of Emi1 synthesis. Cdh1 activity never drops very low, so (presumably) replication origins are continuously relicensed, and DNA synthesis proceeds continuously rather than in discrete rounds of replication.

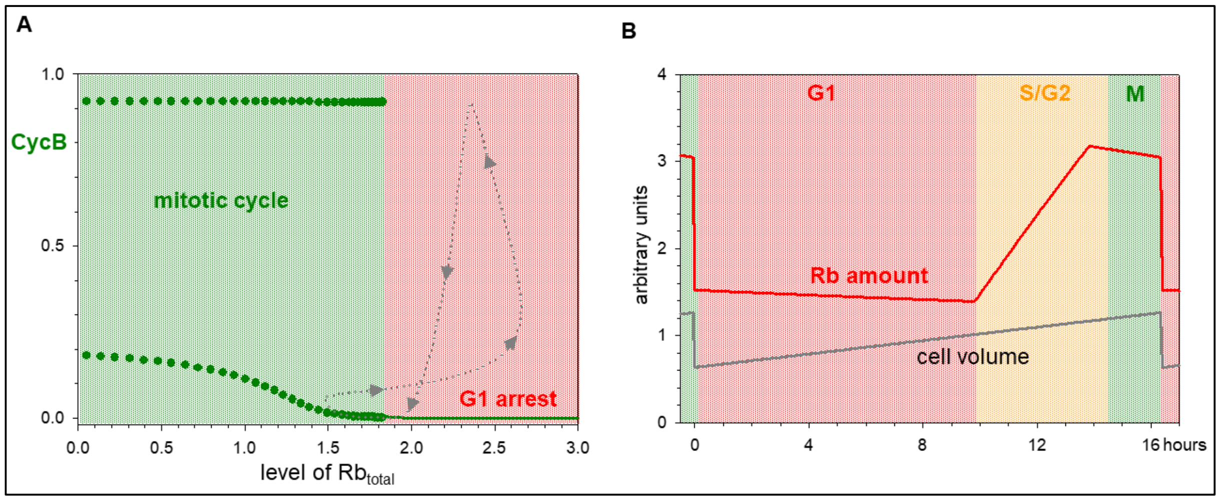

Fig. S5. Checkpoint mechanisms convert spontaneous cell-cycle oscillations into conditional cycles, contingent on execution of certain events. Related to Figure 6. (A) Bifurcation diagram (CycB vs concentration of Rb_{total}) for the cell-growth checkpoint (G1/S transition). Solid green lines: stable steady states; solid green circles: maximum and minimum activity of CycB on a limit-cycle oscillation (spontaneous mitotic cycles). The dashed gray line is the conditional cell cycle, contingent on [Rb_{total}] dilution by cell growth (movement from right to left) and the doubling of total Rb amount (movement from left to right) when the Retinoblastoma protein is synthesized in S phase. The limit cycles arise at a 'homoclinic saddle-loop' bifurcation at [Rb_{total}] ≈ 1.83. **(B)** Temporal changes of Rb amount and cell volume during the cell cycle in the simulation of Figure 6.

Journal of Cell Science • Supplementary information

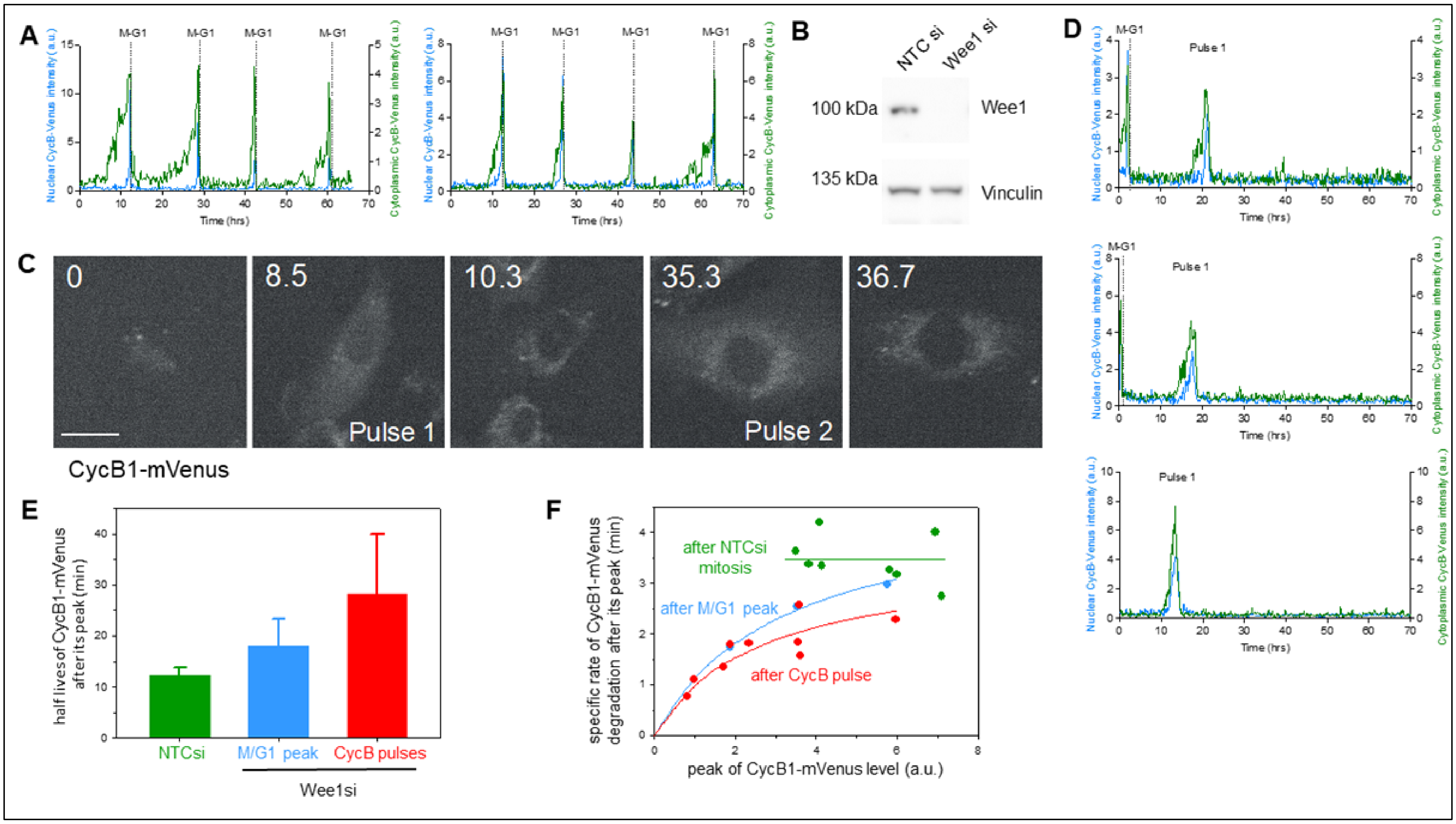

Fig. S6. Cdk1:CycB induces Cdc20-endocycles in the absence of Wee1. Related to Fig. 7. **(A)** Normalised CycB1-mVenus intensity in individual cells treated with control siRNA and undergoing normal mitotic cycles. **(B)** Western blot for Wee1 in NTC- and Wee1-siRNA treated cells. Vinculin is used as a loading control. **(C)** Still images of hTert-RPE1 CycB1-mVenus labelled cells from timelapse experiments. Wee1 was depleted by siRNA 6 h prior to the start of filming (t = 0 h). The cell displays two interphase pulses of CycB1-mVenus expression in the absence of further mitoses. Time shown in hours. Scale bar is 10 µm. **(D)** Normalised CycB1-mVenus intensity in individual cells treated with Wee1 siRNA with one pulse only, plotted from the time of timelapse start (t = 0 h). For A, C and D, n=1 is shown representative of three biological repeats. **(E)** Half-life (min) of CycB1-mVenus after its peak intensity value. **(F)** Specific rate of CycB1-mVenus degradation (min^{-1}) in single cells as a function of preceding CycB1-mVenus intensity peak. For NTC siRNA treated cells the solid line represent the mean vale. In case of Wee1 siRNA treated cells the solid lines are calculated by fitting a hyperbole (spec.rate=a*CycB/(b+CycB) by least square regression.

Journal of Cell Science • Supplementary information

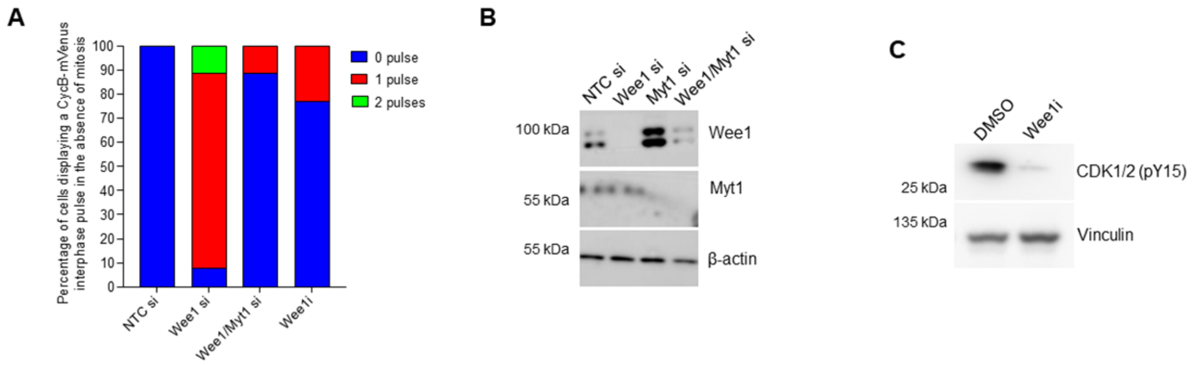

Fig. S7. Cdc20 endocycles are weakened in the complete absence of inhibitory Cdk1 phosphorylation. Related to Fig. 7. (A) Graph to show comparison of Wee1 depleted cells to Wee1/Myt1 co-depleted cells and Wee1 inhibitor (Wee1i, 2.5 μM) treated cells. Wee1/Myt1 and Wee1i treated cells display fewer CycB1-mVenus oscillations than Wee1 only depleted cells. **(B)** Western blot showing co-depletion of Wee1 and Myt1. β-actin is used as a loading control. **(C)** Western blot showing reduction in CDK Y15 phosphorylation after treatment with 2.5 μM Wee1i for 2 h. Vinculin is used as a loading control.

Journal of Cell Science • Supplementary information

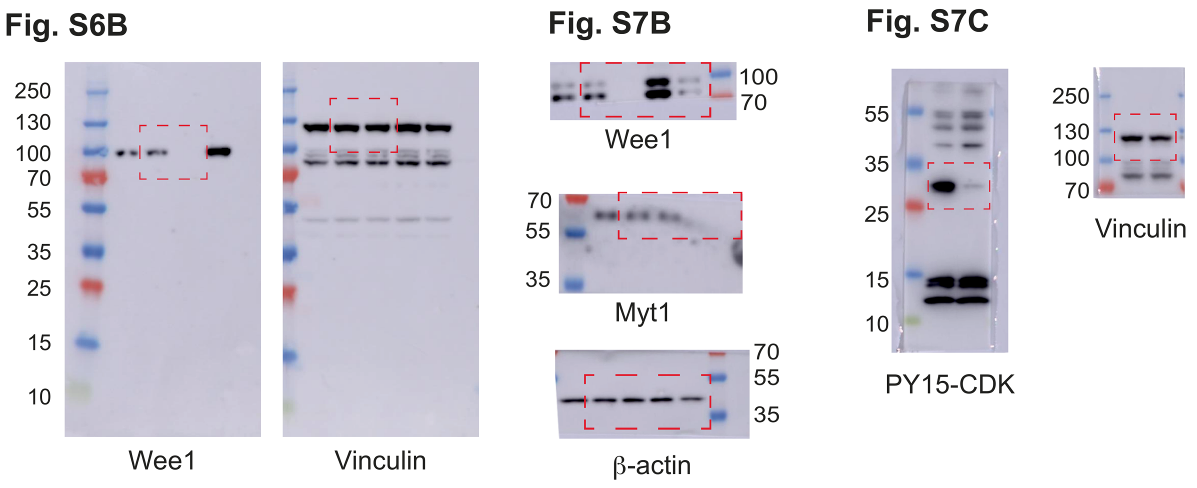

Fig. S8. Uncropped western blots.

Table S1. Kinetic parameters of the mammalian cell model. Rate constants (*k's*) have a dimension of h^{-1} while other parameters are dimensionless.

CycE synthesis/degradation	k_{scyce}=1.5, k_{dcyce}'=0.6, k_{dcyce}"=1.5
CycA synthesis/degradation	k_{scyca}=0.45, k_{dcyca}'=0.045, k_{dcyca}"=0.75, k_{dcyca}=3.75
E2F phosphorylation/dephosphorylation	$E2F_T$=1, k_{dpe2f}=0.3, k_{pe2f}=1.5, K_{drbe2f}=0.001
Rb phosphorylation/dephosphorylation	J_{Rb}=0.1, k_{prb}=15, k_{dprb}=10.5, α=1, CycD=1
Emi1 synthesis/degradation	k_{semi1}=1.5, k_{demi1}'=0.15, k_{demi1}"=4.5, k_{demi1}=7.5, K_{dc1e1}=0.01
CycB synthesis/degradation	k_{scycb}=0.3, k_{dcycb}'=0.06, k_{dcycb}"=0.75, k_{dcycb}=3.75
CycB dephosphorylation (CDK1)	k_{pyme}'=0, k_{pyme}=30, k_{dpyme}=6, J_{yme}=0.1, k_{25}'=0.45, k_{25}=15, k_{wee}'=0.15, k_{wee}=15
Cdh1 phosphorylation	$Cdh1_{tot}$=1, k_{acdh1}=15, k_{icdh1}'=15, k_{icdh1}"=30, k_{icdh1}=6000
Cdc20 phosphorylation	k_{icdc20}=15, k_{acdc20}=3, ε=1, SAC=1
Polo synthesis/degradation	k_{spolo}'=0.15, k_{dpolo}'=0.15, k_{dpolo}"=15
Polo phosphorylation	k_{apolo}'=4.5, k_{apolo}"=15, k_{ipolo}=7.5, J_{polo}=0.01
ENSA phosphorylation	k_{GwENSA}=15, $ENSA_{tot}$=4, k_{catB55}=15
Gwl phosphorylation	k_{ppx}'=6, k_{CdkGwl}=30, k_{B55Gwl}=60, Gw_{tot}=1
PP2A:B55 – ENSA complex formation	$B55_{tot}$=1, k_{ass}=7500, k_{diss}=4.5
Rbtot synthesis/degradation	k_{srb}=0.02 or 0.1, k_{drb}=0.023
Volume growth rate	μ=0.0385 h^{-1}

Journal of Cell Science • Supplementary information

Table S2. XPPAUT code for simulation of the mammalian cell cycle model.

```
# XPPAut model for the human cell cycle
# Differential equations
CycE' = kscyce*E2F - (kdcyce' + kdcyce"*CycA)*CycE
CycA' = kscyca*E2F - (kdcyca' + kdcyca"*Cdc20 + kdcyca*Cdh1)*CycA
E2FPt' = kpe2f*(CycA+eps*Cdk1)*(E2FT - E2FPt) - kdpe2f*E2FPt
Rb' = kdprb*(Rbt-Rb)/(Jrb+Rbt-Rb) - kprb*(CycE+CycA+eps*Cdk1)*Rb/(Jrb+Rb)
Emi1' = ksemi1*E2F - (kdemi1' + kdemi1"*Cdh1 + kdemi1*Polo)*Emi1
CycB' = kscycb*CycA - Vdcycb*CycB
Cdk1' = kscycb*CycA + V25*(CycB - Cdk1) - Vwee*Cdk1 - Vdcycb*Cdk1
Cdh1' = kacdh1*(Cdh1t-Cdh1) - (kicdh1'*CycE+kicdh1"*CycA+kicdh1*eps*Cdk1)*Cdh1
Cdc20' = kacdc20*eps*Cdk1*(1-Cdc20) - kicdc20*PP2AB55*Cdc20
PoloT' = kspolo' - (kdpolo' + kdpolo"*Cdh1)*PoloT
Polo' = (kapolo'*CycA + kapolo"*eps*Cdk1)*(PoloT-Polo)/(Jpolo+PoloT-Polo)-kipolo*Polo/(Jpolo+Polo)
pENSAt' = kGwENSA*pGwl*(ENSAtot - pENSAt) - kcatB55*Complex
pGwl' = kCdkGwl*eps*Cdk1*(Gwtot - pGwl) - (kppx' + kB55Gwl*PP2AB55)*pGwl
PP2AB55' = (kdiss + kcatB55)*Complex-kass*(pENSAt-Complex)*(B55tot-Complex)
# Algebraic equations
Rbt = Rbtot/(1 + alpha*CycD)
BB1 = Rb + E2FT + Kdrbe2f
RbE2F = (BB1 - sqrt(BB1^2 - 4*Rb*E2FT))/2
E2F = (E2FT-E2FPt)*(E2FT-RbE2F)/E2FT
BB2 = Emi1 + Cdh1tot + Kdc1e1
Cdh1Emi1 = (BB2 - sqrt(BB2^2 - 4*Emi1*Cdh1tot))/2
Cdh1t = Cdh1tot - Cdh1Emi1
YMEP = GK(kpyme'*CycA+kpyme*eps*Cdk1,kdpyme,Jyme,Jyme)
V25 = k25' + k25*YMEP
Vwee = kwee' + kwee*(1 - YMEP)
Vdcycb = kdcycb' + kdcycb"*Cdc20 + kdcycb*Cdh1
Complex = B55tot-PP2AB55
# Auxiliary variables
aux E2F = (E2FT-E2FPt)*(E2FT-RbE2F)/E2FT
aux Cdh1t = Cdh1tot - Cdh1Emi1
# Goldbeter-Koshland function
GB(arg1,arg2,arg3,arg4) = arg2-arg1+arg2*arg3+arg1*arg4
GK(arg1,arg2,arg3,arg4) = 2*arg1*arg4/(GB(arg1,arg2,arg3,arg4)+sqrt(GB(arg1,arg2,arg3,arg4)^2-4*(arg2-
arg1)*arg1*arg4))
# Parameter values
p kscyce=1.5, kdcyce'=0.6, kdcyce"=1.5
p kscyca=0.45, kdcyca'=0.045, kdcyca"=0.75, kdcyca=3.75
p Rbtot=1.75, JRb=0.1, kprb=15, kdprb=10.5, alpha=1, CycD=1
p E2FT=1, kdpe2f=0.3, kpe2f=1.5, Kdrbe2f=0.001
p ksemi1=1.5, kdemi1'=0.15, kdemi1"=4.5, kdemi1=7.5, Kdc1e1=0.01
p Cdh1tot=1, kacdh1=15, kicdh1'=15, kicdh1"=30, kicdh1=6000
p kscycb=0.3, kdcycb'=0.06, kdcycb"=0.75, kdcycb=3.75
p kpyme'=0, kpyme=30, kdpyme=6, Jyme=0.1, k25'=0.45, k25=15, kwee'=0.15, kwee=15
p kicdc20=15, kacdc20=3, eps=1, SAC=1
p kspolo'=0.15, kdpolo'=0.15, kdpolo"=15
p kapolo'=4.5, kapolo"=15, kipolo=7.5, Jpolo=0.01
p ENSAtot=4, B55tot=1, kass=7500, kdiss=4.5, kcatB55=15
p kGwENSA=15, kppx'=6, kCdkGwl=30, kB55Gwl=60, Gwtot=1
# XPP settings
@ METH=stiff,XLO=0,XHI=50,YLO=0,YHI=1.6,total=50,dt=0.05,XP=time
@ NPLOT=8,YP=CycE,YP2=CycA,YP3=Emi1,YP4=CycB,YP5=Cdk1,YP6=Cdh1,YP7=Cdc20,YP8=Rb
done
```

Journal of Cell Science • Supplementary information

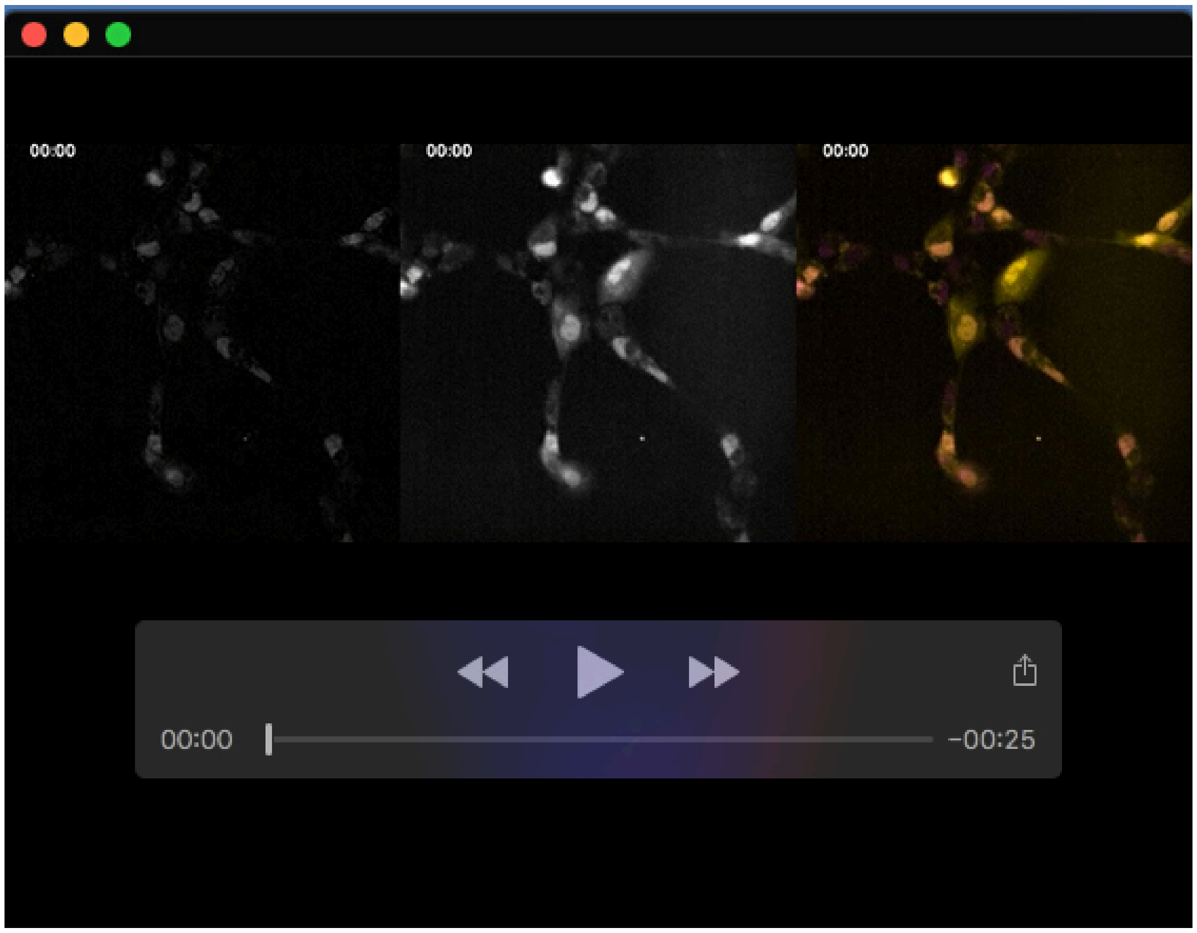

Movie 1. Cyclin A2-mVenus mRuby-PCNA RPE1 cells treated with DMSO. Left is mRuby-PCNA, middle is CyclinA2-mVenus and right is merged image (mRuby-PCNA in magenta, CyclinA2-mVenus in yellow).

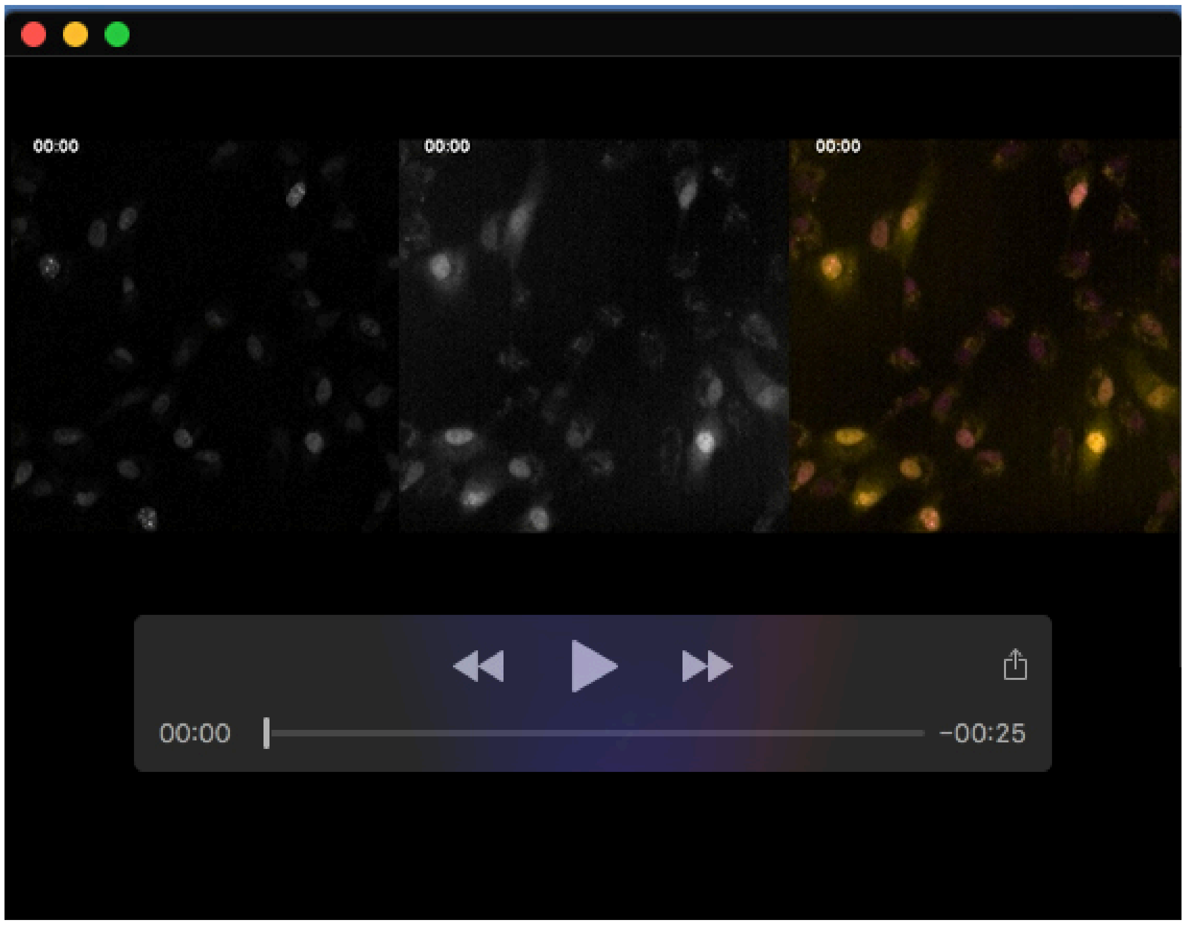

Movie 2. Cyclin A2-mVenus mRuby-PCNA RPE1 cells treated with 7.5 μm CDK1i. Left is mRuby-PCNA, middle is CyclinA2-mVenus and right is merged image (mRuby-PCNA in magenta, CyclinA2-mVenus in yellow).

Journal of Cell Science • Supplementary information

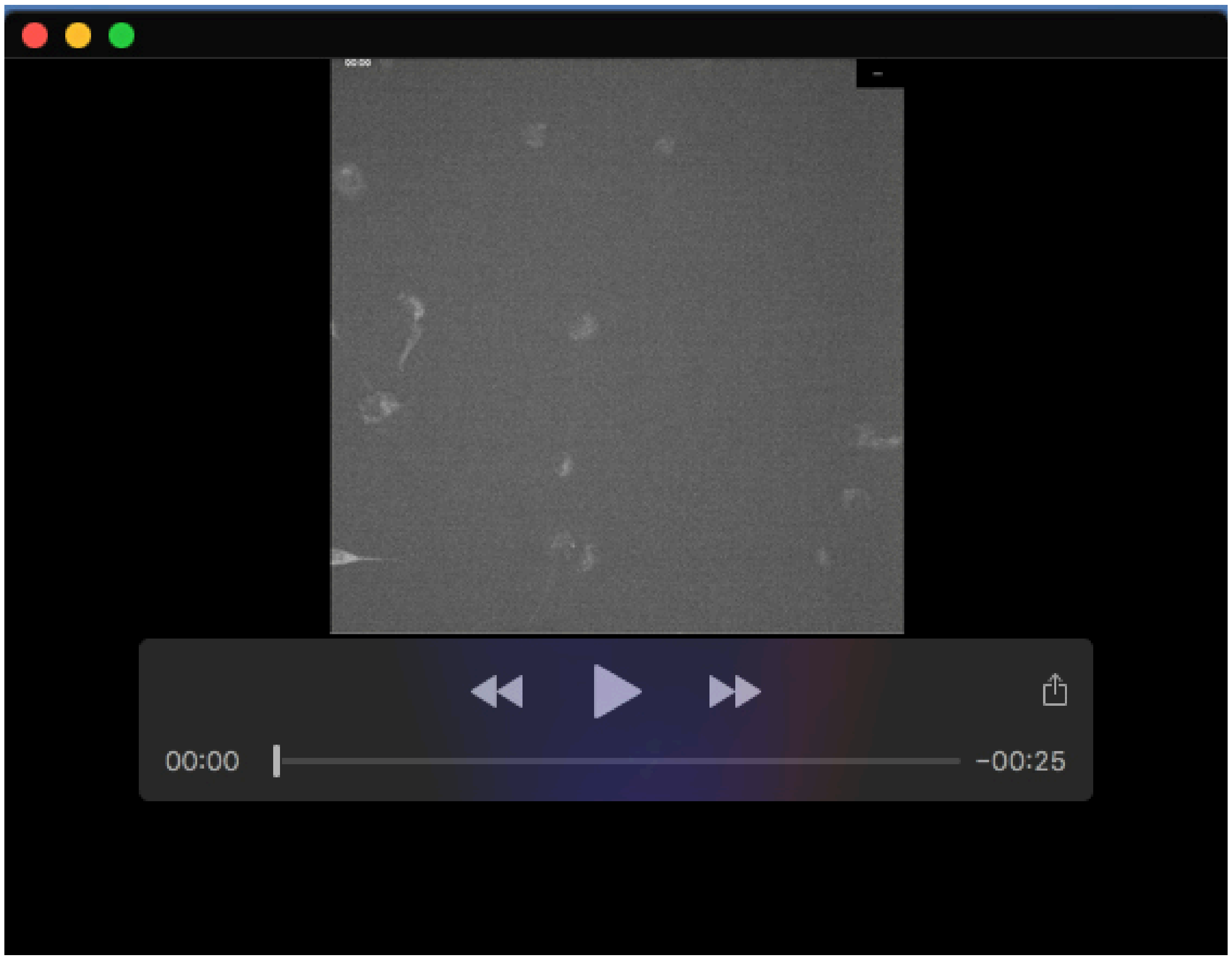

Movie 3. Cyclin B1-mVenus RPE1 cells transfected with NTC siRNA.

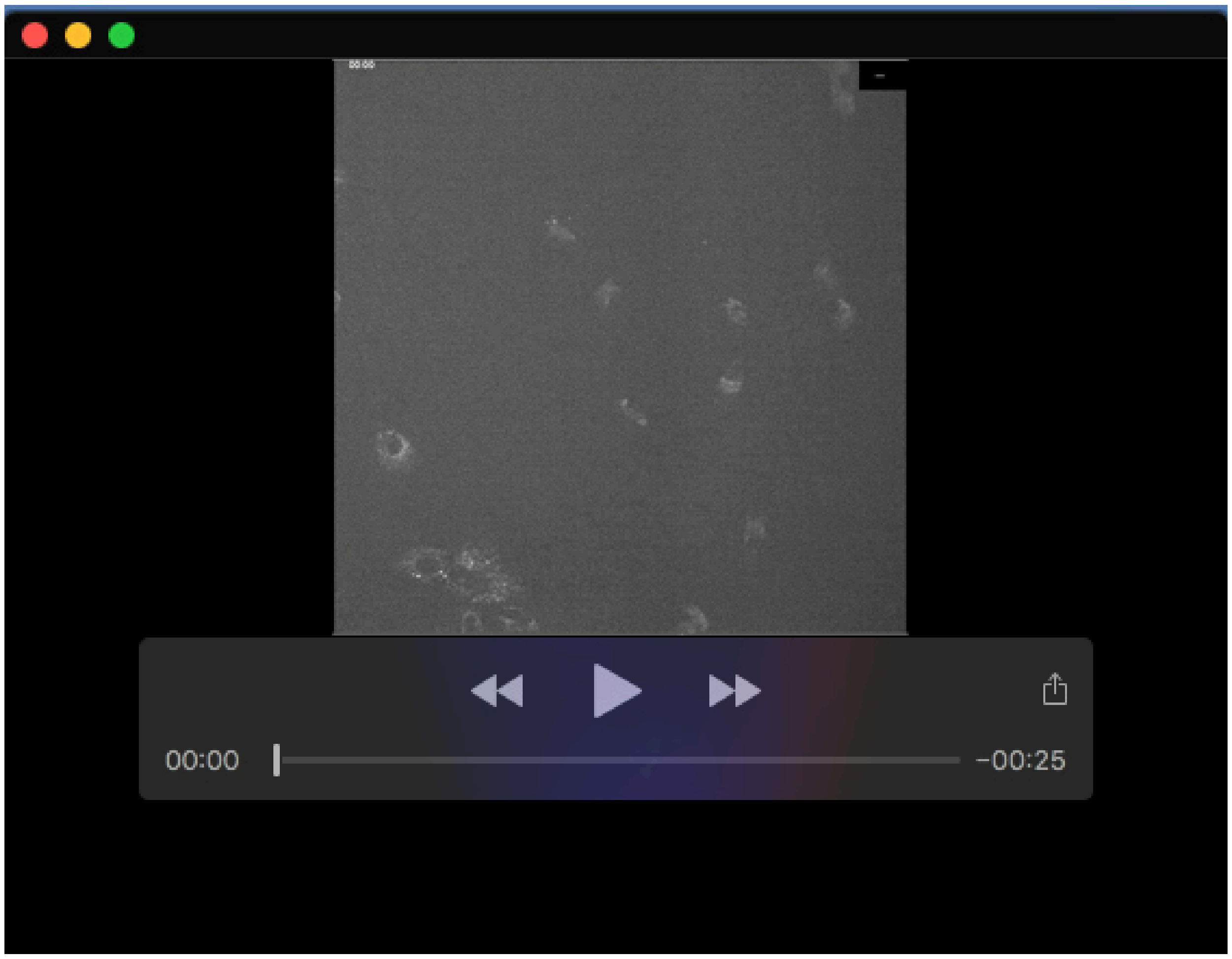

Movie 4. Cyclin B1-mVenus RPE1 cells transfected with Wee1 siRNA.

Journal of Cell Science • Supplementary information
