# Supplementary material for: The oscillation of mitotic kinase governs cell cycle latches in mammalian cells
Source: J Cell Sci. 2024 Feb 13;137(3):jcs261364. doi: 10.1242/jcs.261364 (PMC10911285; doi:10.1242/jcs.261364)
Supplement: Supplementary file 1 [file joces-137-261364_review_history.pdf]

Journal of Cell Science

The oscillation of mitotic kinase governs cell cycle latches in mammalian cells

Calin-Mihai Dragoi, Ekjot Kaur, Alexis R. Barr, John J. Tyson and Béla Novák
DOI: 10.1242/jcs.261364

Editor: David Stephens

Review timeline
Original submission: 26 May 2023
Editorial decision: 19 July 2023
First revision received: 23 November 2023
Accepted: 29 December 2023

Original submission

First decision letter

MS ID#: JOCES/2023/261364

MS TITLE: The oscillation of mitotic kinase governs cell cycle latches in mammalian cells

AUTHORS: Calin-Mihai Dragoi, Alexis R Barr, John J Tyson, and Bela Novak

ARTICLE TYPE: Research Article

We have now reached a decision on the above manuscript.

To see the reviewers' reports and a copy of this decision letter, please go to: https://submit-jcs.biologists.org and click on the 'Manuscripts with Decisions' queue in the Author Area. (Corresponding author only has access to reviews.)

As you will see, the reviewers raise a number of substantial criticisms that prevent me from accepting the paper at this stage. They suggest, however, that a revised version might prove acceptable, if you can address their concerns. If you think that you can deal satisfactorily with the criticisms on revision, I would be pleased to see a revised manuscript. We would then return it to the reviewers.

In particular, the reviewers highlight the need to address additional controls, provide further validation, and better explain the model in context of the existing literature.

I am sorry that this has taken some time to review and would also like to point out that the reviewer apologizes for the typos in their text.

Please ensure that you clearly highlight all changes made in the revised manuscript. Please avoid using 'Tracked changes' in Word files as these are lost in PDF conversion.

I should be grateful if you would also provide a point-by-point response detailing how you have dealt with the points raised by the reviewers in the 'Response to Reviewers' box. Please attend to all of the reviewers' comments. If you do not agree with any of their criticisms or suggestions please explain clearly why this is so.

© 2024. Published by The Company of Biologists under the terms of the Creative Commons Attribution License (https://creativecommons.org/licenses/by/4.0/).

Reviewer 1

Advance summary and potential significance to field

In this study the authors have modelled the human cell cycle using the published literature and some new experimental data as the basis for their model. The oscillation of CDK cycles through APC/C-CDC20 inactivation of mitotic cyclins, and the requirement of CDH1 to repress mitotic cyclins to establish a G1 phase are well established; thus, the potential advance described in this study is modelling endo-cycles. Thus, the accuracy and utility of the model depends on the authors' interpretation and reading of the literature, and in this respect I have a number of criticisms that in my view preclude publication of this study in its present form.

Comments for the author

Major point:
The authors have been selective in the papers that they cite; in particular, they base most of their modelling on Pomerening at al., 2008. They have not taken into account the results from Andrew Porter on cells expressing CDK1-AF (DOI: 10.1074/jbc.M607910200 and doi.org/10.1093/hmg/ddt133), nor do they discuss those of Philip Kaldis (doi.org/10.1038/s41388-018-0464-0). The authors' interpretation of their own experiments using siRNA to deplete WEE1 to induce a constitutively active CDK1 do not take into account that MYT1 is still present in the cell, which also inactivates Cyclin B-CDK1 by phosphorylation on T14 and Y15.
In these experiments the authors ascribe bursts of Cyclin B increase to CDC20 endo-cycles but do not test this, for example by adding APC/C inhibitors to the cells.
In summary, I find that there are too many over-simplifications and too few experiments to control for alternative explanations to support the model presented.

Minor points:
1) Page 2, line 3: mitosis is not just the alignment of chromosomes on the spindle: I suspect the authors mean metaphase here.
2) Page 3, line 12: it is inaccurate to say that animal cells normally arrest soon after birth. Some cells do many others (embryonic cells, stem cells) do not.
3) Page 2, line 15: it is questionable whether the Restriction Point is a checkpoint. It certainly does not fit the original Hartwell and Weinert definition of a checkpoint because the initiation of a late event is not dependent on the completion of an early event (DOI: 10.1126/science.2683079). The Restriction Point is more accurately described as a control point.
4) Page 7, last paragraph: EMI1 depletion causing endoreduplication was shown by Di Fiore et al coincidentally with Machida and Dutta but is not referenced.

Reviewer 2

Advance summary and potential significance to field

Here Novak and colleagues present a moderately-complicated, moderately-realistic model of the mammalian cell cycle and use it to help explain various ways that endocycles can emerge. I found modeling to be interesting and relatively easy to understand. This is accompanied by experimental studies that examine whether the predicted endocycles do occur.

Comments for the author

. I was not completely convinced by this part of the paper; I think the authors need to strengthen it with more cell tracking data, alternative probes, and some Wee1 inhibitor studies.

Major points:

1. Fig 4: It is nice to see experiments that test some of the predictions that come out of the model, but the experiments in Fig 4 on endocycles could be more thorough and convincing. First, I cannot see the typical replication foci one expects from the PCNA sensor (Fig 4D; see Leonhardt J Cell Biol 2000, for comparison), which makes me less than completely convinced that the authors can accurately judge the time of S phase entry and exit. Perhaps a live-cell DNA dye could

© 2024. Published by The Company of Biologists under the terms of the Creative Commons Attribution License (https://creativecommons.org/licenses/by/4.0/).

strengthen the conclusion that these are real S phases. The Spencer Cdk1 sensor might be helpful to use as well.

2. Fig 4: Moreover, given the variability of the results, ideally one would plot time courses for many individual cells, not just a few. It would also be good to present videos of a few typical cells.

3. Fig 4: And finally, are there any signs of M-phase—e.g. rounding up, chromatin condensation, nuclear envelope breakdown—in the endocycling cells? In other words, are we sure they are failing to enter mitosis as opposed to failing to carry out anaphase or cytokinesis?

4. Fig 7: There are good small molecule Wee1 inhibitors available. How do their effects compare with the Wee1 knockdown phenotype?

Minor points:

1. The model is intermediate in its complexity. It includes more than the bare bones circuitry needed to model interconnected M-phase and S-phase switches/latches, but much less than what we know the real circuit to be like. This is fine, but the authors should acknowledge/explain this.

2. One example of this sort of simplification is the authors' treatment of multisite phosphorylation reactions, which are commonplace in cell cycle regulation, as single-site processes approximated by Goldbeter-Koshland functions. The authors should say why they did this (I think is that it greatly reduces the number of time-dependent species in the model but still yields the sigmoidal response functions that help generate switches and relaxation oscillations, and not that they know that any of these reactions are running near saturation).

3. I suggest that the authors discuss the different time scales of the model. For example, if I understand it correctly, on a time scale of minutes one can regard the system as bistable, with alternative stable M-phase and G1-phase states and snappy transitions between them, whereas on a longer time scale the system is a relaxation oscillator, marching along the S-shaped nullclines because of the slow accumulation of CycE (during G1 -> M) or Cdc20 (during M -> G1). These two-time scale relaxation oscillators are seen in many contexts in biology; in fact, I cannot think of a biological phenomenon driven by a Repressilator/Goodwin-type, one-time scale oscillator, even though they have been important to the development of the field.

4. Some acknowledgement of the many other mathematical models of the mammalian cell cycle, and a description of the differences and similarities between the authors' model and these models, is essential.

5. I do not find it helpful to include negative concentrations on the bifurcation diagrams. One can see the difference between an irreversible and a hysteretic response curve just as easily without including the nonphysical domain of the function.

6. A lot of the modeling is presented very simply, which I applaud. But on the bifurcation diagrams, it would be helpful to the novice if the authors were to point out the basics that are skipped over here: E.g., for the values of the bifurcation parameter where there are three steady states, the upper and lower states are stable and the middle state is unstable. And when the bifurcation parameter exceeds a critical value (at the saddle-node bifurcation), the system becomes monostable—there is a single steady-state, and it is stable. And so if CycE (or Cdc20) is changing slowly, the trajectory will cling to one leg of the S-shaped curve for as long as it can, and then plummet down (for the CycE bifurcation diagram) or explode up (for Cdc20) to the other leg.

First revision

<u>Author response to reviewers' comments</u>

Reply to Reviewers

© 2024. Published by The Company of Biologists under the terms of the Creative Commons Attribution License (https://creativecommons.org/licenses/by/4.0/).

Reviewer 1 Advance Summary and Potential Significance to Field:
In this study the authors have modelled the human cell cycle using the published literature and some new experimental data as the basis for their model. The oscillation of CDK cycles through APC/C-CDC20 inactivation of mitotic cyclins, and the requirement of CDH1 to repress mitotic cyclins to establish a G1 phase are well established; thus, the potential advance described in this study is modelling endo-cycles. Thus, the accuracy and utility of the model depends on the authors' interpretation and reading of the literature, and in this respect I have a number of criticisms that in my view preclude publication of this study in its present form.

Reviewer 1 Comments for the Author:
Major point:
The authors have been selective in the papers that they cite; in particular, they base most of their modelling on Pomerening at al., 2008. They have not taken into account the results from Andrew Porter on cells expressing CDK1-AF (DOI: 10.1074/jbc.M607910200 and doi.org/10.1093/hmg/ddt133), nor do they discuss those of Philip Kaldis (doi.org/10.1038/s41388-018-0464-0).
We acknowledge that the perturbation of CDK1 inhibitory phosphorylation on Thr14/Tyr15 may result in a broad range of altered cell cycle phenotypes, as indeed shown by Pomerening et al. 2008, as well as by the papers mentioned by the referee. We are presently aiming to address the mechanisms by which CDK1 perturbations may result in the bypass of certain cell cycle phases, rather than an exhaustive list of effects such perturbations might produce. Nevertheless, have explicitly included considerations of the altered phenotypes observed by Porter's and Kaldis's groups, as requested by the referee (see lines: 276, 347-348, 448-449 and references)

The authors' interpretation of their own experiments using siRNA to deplete WEE1 to induce a constitutively active CDK1 do not take into account that MYT1 is still present in the cell, which also inactivates Cyclin B-CDK1 by phosphorylation on T14 and Y15.
First of all, the activity of Wee1 in the model represents the cumulative activity of Wee1 & Myt1, which we forgot to mention in the original submission, but their joint activity is now stated clearly (see line: 350). In addition, we performed experiments where we co-depleted Wee1 and Myt1 by siRNA. Co-depletion of Myt1 with Wee1 generated a different phenotype to that of Wee1 depletion alone where only 11.5% of cells displayed one pulse of CycB-mVenus in the absence of mitosis. We now include this data in Supplementary Figure 7A-C and in results (lines 445-449). The most common phenotype in Wee1/Myt1 co-depleted cells was that they completed an abnormal mitosis (88.5% of cells) where cells remained rounded up in a mitotic arrest for long periods of time (>1h) and then frequently failed cytokinesis to become multinucleate. This phenotype was very similar to that observed using the Wee1 inhibitor, MK1775 (see also comments below to Reviewer 2).

In these experiments the authors ascribe bursts of Cyclin B increase to CDC20 endo-cycles but do not test this, for example by adding APC/C inhibitors to the cells.
We agree with the reviewer that this is an important point and one which we were keen to test. However, we ran into several difficulties in testing the dependence of CycB pulses on Cdc20 activity.

First, we tried to co-deplete Wee1 and Cdc20 (Figure R1A) which, if our model is correct, should abolish CycB bursts. When we depleted Cdc20 by siRNA, we saw no change in CycB burst frequency (Figure R1B). However, we also saw no change in mitotic length (Figure R1C), suggesting that our depletion of Cdc20 was insufficient. This is consistent with work described in Varetti et al., Mol Cell 2011, Baumgarten et al., Cell Cycle 2009, and Wolthuis et al., Mol Cell 2008, where they show that even 5% of remaining Cdc20 protein can maintain its function in cells.

Second, we tried APC/C inhibitors. There are two available - proTAME thought to target both Cdc20 and Cdh1, and APCin thought to be more Cdc20 specific. At high concentrations (6 mm ProTAMe and 25 mm APCin), we observed an extended duration of mitosis, suggesting that these inhibitors were active in cells (Figure R1C).
However, this activity was not prolonged and 500 mins (~8 h) after adding inhibitors to cells, we could see the inhibitory effect declining (Figure R1D). Therefore, we were unable to use these inhibitors in our long (72h) time-course experiments.

© 2024. Published by The Company of Biologists under the terms of the Creative Commons Attribution License (https://creativecommons.org/licenses/by/4.0/).

Figure R1. A. Western blot showing Wee1 and Cdc20 co-depletion. **B.** Graph showing number of CycB interphase pulses in Wee1 depleted cells compared to Wee1 and Cdc20 co-depleted cells. **C.** Length of mitosis (NEBD to anaphase) in cells treated with Wee1si, Wee1si and Cdc20si, or Wee1si and APC/Ci. **D.** Correlation between time of mitotic entry (relative to APC/Ci addition at t = 0 min) and length of mitosis. Red dashed line represents when mitotic lengths start to decline suggesting that the stability of the APC/Ci in the media may be short-lived.

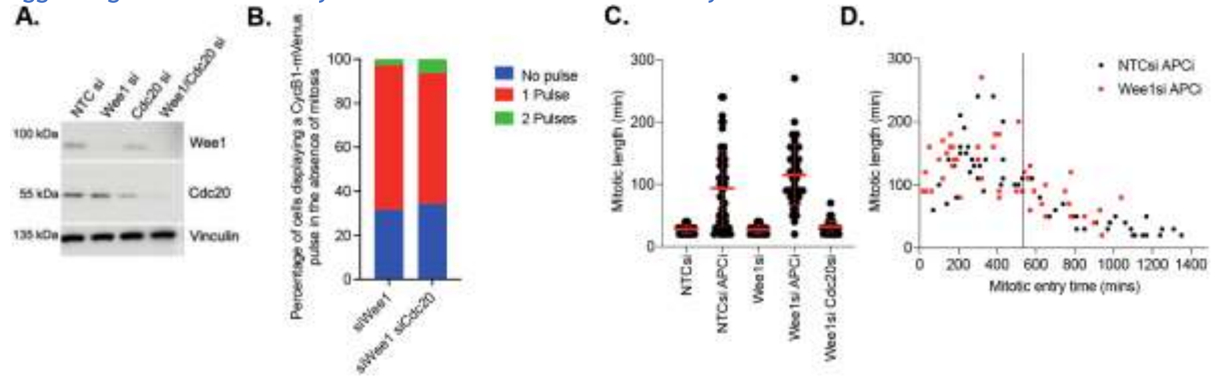

We have chosen not to include these data in the paper but wanted to show the reviewers why we were unable to test these model predictions. In the absence of a direct experimental test of the dependence of CycB pulses on APC/C:Cdc20 activity, we have analysed the kinetics of CycB degradation during normal mitotic exit (M/G1 peaks) and during CycB pulses in Wee1si treated cells (see lines 428-444). We have estimated the specific rate of CycB degradation in single cells, which is the inverse of its half-life ($k = \ln 2/t_{½}$). We found that the specific rate of degradation shows the same hyperbolic dependence on the maximum CycB level after APC/C:Cdc20-dependent M/G1 peaks and after CycB pulses in Wee1si treated cells. This kinetic analysis suggests that the interphase CycB pulses are indeed Cdc20 endocycles.
We have reworded the text in the results (line 428-444) and discussion (lines 552-556) to state the limitations of this study and that, while our current experimental data are consistent with the model predictions, they are not definitive proof of Cdc20's role in CycB pulses.

In summary, I find that there are too many over-simplifications and too few experiments to control for alternative explanations to support the model presented.
(1) Every 'model' is a simplification of 'reality'. At issue here is whether our particular simplification is appropriate to understand irreversible progression through a normal cell cycle and reversible endocycles, without introducing additional complications that are not relevant to the question at hand.
(2) We trust our additional experiments and calculations will address the second criticism.

Minor points:
1) Page 2, line 3: mitosis is not just the alignment of chromosomes on the spindle: I suspect the authors mean metaphase here.
Line 32: mitosis is replaced with metaphase.
2) Page 3, line 12: it is inaccurate to say that animal cells normally arrest soon after birth. Some cells do, many others (embryonic cells, stem cells) do not.
Line 42-43: we have narrowed down the statement by 'Most somatic cells in animals arrest soon after birth (in G1 phase of the cycle,…'
3) Page 2, line 15: it is questionable whether the Restriction Point is a checkpoint. It certainly does not fit the original Hartwell and Weinert definition of a checkpoint because the initiation of a late event is not dependent on the completion of an early event (DOI: 10.1126/science.2683079). The Restriction Point is more accurately described as a control point.
Line 41-42: we use a broader definition for checkpoints 'as pauses in cell cycle progression, lasting until specific physiological conditions are satisfied.'
4) Page 7, last paragraph: EMI1 depletion causing endoreduplication was shown by Di Fiore et al coincidentally with Machida and Dutta but is not referenced.
line 324: True, the Di Fiore at al. reference is added.
*****
Reviewer 2 Advance Summary and Potential Significance to Field:
Here Novak and colleagues present a moderately-complicated, moderately-realistic model of the mammalian cell cycle and use it to help explain various ways that endocycles can emerge. I found

© 2024. Published by The Company of Biologists under the terms of the Creative Commons Attribution License (https://creativecommons.org/licenses/by/4.0/).

modeling to be interesting and relatively easy to understand. This is accompanied by experimental studies that examine whether the predicted endocycles do occur.
Thanks for these nice words.

Reviewer 2 Comments for the Author: .
I was not completely convinced by this part of the paper; I think the authors need to strengthen it with more cell tracking data, alternative probes, and some Wee1 inhibitor studies.

Major points:
1. Fig. 4: It is nice to see experiments that test some of the predictions that come out of the model, but the experiments in Fig 4 on endocycles could be more thorough and convincing. First, I cannot see the typical replication foci one expects from the PCNA sensor (Fig 4D; see Leonhardt J Cell Biol 2000, for comparison), which makes me less than completely convinced that the authors can accurately judge the time of S phase entry and exit. Perhaps a live-cell DNA dye could strengthen the conclusion that these are real S phases. The Spencer Cdk1 sensor might be helpful to use as well.
We apologise for the quality of the images, they were not as clear as they should have been. We have now updated the images in Figure 4E and Figure S3E to demonstrate how we can easily detect PCNA foci and S- phase entry and exit using our mRuby-PCNA marker in cells. Of note, we have used mRuby-PCNA routinely to judge S-phase entry and exit timing (Barr et al., Nat Comms 2017; Pennycook & Barr, Open Biol 2021; Crozier*, Foy*, Adib* et al., Mol Cell 2023). Together with the inclusion of new Supplementary Movies 1-4 (Movies 1 and 2 are CyclinA2-mVenus, mRuby-PCNA expressing cells) and FACS plots (Figure S3B) showing discrete 8n and 16n peaks (indicative of real S-phases and complete DNA replication) to accompany Figure S3A, we hope that this reviewer is now convinced that we can easily detect S-phase entry and exit using this system.
We do not include a live cell DNA dye in our assays as we find that inclusion of SiR-DNA impacts cell viability over the long imaging periods (72h) used here. This has also been reported elsewhere (Sen et al., Sci Rep 2018; Rajendraprasad et al., Life Sci Alliance 2023).

2. Fig. 4: Moreover, given the variability of the results, ideally one would plot time courses for many individual cells, not just a few. It would also be good to present videos of a few typical cells.
We agree that it would be ideal/wonderful to plot time courses for many individual cells. However, in these particular experiments this is technically very difficult for two reasons:

i) due to cell migration and the long imaging periods required to visualise pulses (72h) we have very few cells per field that remain in the field of view for the whole imaging period. Therefore, automated tracking detects many short tracks where cells migrate out of the field of view;
ii) automated tracking of cells over 72h periods is not yet reliable and therefore we manually tracked cells in ImageJ to ensure 100% reliability of tracking and quantification, this obviously limits the number of tracks that we can extract as this is very time-consuming.

To address this comment, we have included representative videos of typical cells (Supplementary movies 1-4) that we hope, along with the quantification of phenotypes already given will show that our manually tracked data is representative of the cells imaged. We have also stated why cells were manually tracked in the methods (lines 713-714 and 724-726).

3. Fig. 4: And finally, are there any signs of M-phase—e.g. rounding up, chromatin condensation, nuclear envelope breakdown—in the endocycling cells? In other words, are we sure they are failing to enter mitosis as opposed to failing to carry out anaphase or cytokinesis?
In our control movies we can detect rounding up and nuclear envelope breakdown with the mRuby-PCNA marker that we use (unfortunately we cannot detect chromatin condensation). However, we detect no signs of mitotic rounding or nuclear envelope breakdown in the endocycling cells and so we are certain that the cells are failing to enter mitosis. This should now be clear to readers from our inclusion of the Supplementary Movies, in particular Supplementary Movie 2 (CyclinA2-mVenus mRuby-PCNA cells treated with CDK1i).
4. Fig. 7: There are good small molecule Wee1 inhibitors available. How do their effects compare with the Wee1 knockdown phenotype?
We performed experiments with the Wee1i (MK1775). Inhibition of Wee1 generated a different

© 2024. Published by The Company of Biologists under the terms of the Creative Commons Attribution License (https://creativecommons.org/licenses/by/4.0/).

phenotype to that of Wee1 depletion where only 23.2% of cells displayed one pulse of CycB-mVenus in the absence of mitosis. We now include this data in Supplementary Figure 7A-C and in results (lines 445-449). Instead, the most common phenotype in Wee1 inhibitor treated cells was that cells completed an abnormal mitosis (76.8% of cells) where cells remained rounded up in a state of mitotic arrest for long periods of time (>1h) and then frequently failed cytokinesis to become multinucleate. This phenotype was very similar to that observed when we co-depleted Wee1 and Myt1 (see also comments above to Reviewer 1).

Minor points:
1. The model is intermediate in its complexity. It includes more than the bare bones circuitry needed to model interconnected M-phase and S-phase switches/latches, but much less than what we know the real circuit to be like. This is fine, but the authors should acknowledge/explain this.
We have addressed this issue in line 157-165.
2. One example of this sort of simplification is the authors' treatment of multisite phosphorylation reactions, which are commonplace in cell cycle regulation, as single-site processes approximated by Goldbeter-Koshland functions. The authors should say why they did this (I think is that it greatly reduces the number of time- dependent species in the model but still yields the sigmoidal response functions that help generate switches and relaxation oscillations, and not that they know that any of these reactions are running near saturation).
This issue is addressed during the model description of the Materials and methods in line 634-640.
3. I suggest that the authors discuss the different time scales of the model. For example, if I understand it correctly, on a time scale of minutes one can regard the system as bistable, with alternative stable M-phase and G1-phase states and snappy transitions between them, whereas on a longer time scale the system is a relaxation oscillator, marching along the S-shaped nullclines because of the slow accumulation of CycE (during G1 -> M) or Cdc20 (during M -> G1). These two-time scale relaxation oscillators are seen in many contexts in biology;
Correct, we have expanded the section of 'Mapping the Cell Cycle Clock with Bifurcation Curves' accordingly in line 235-245.
in fact, I cannot think of a biological phenomenon driven by a Repressilator/Goodwin-type, one-time scale oscillator, even though they have been important to the development of the field.
We disagree, because most models of eukaryotic circadian rhythms are Goodwin-type oscillators. See Gonze & Ruoff, Acta Biotheor 69:857, 2021; Kim, IET Syst Biol 10:125, 2016; Kim & Forger, Mol Syst Biol 8:630, 2012; Goldbeter, Proc Roy Soc Biol Sci 261:319, 1995.
4. Some acknowledgement of the many other mathematical models of the mammalian cell cycle, and a description of the differences and similarities between the authors' model and these models, is essential.
We have addressed this issue in line 493-513.
5. I do not find it helpful to include negative concentrations on the bifurcation diagrams. One can see the difference between an irreversible and a hysteretic response curve just as easily without including the nonphysical domain of the function.
We think the negative concentration regime on the bifurcation diagram is important to illustrate the continuous changes caused by genetic and physiological perturbations.
6. A lot of the modeling is presented very simply, which I applaud.
But on the bifurcation diagrams, it would be helpful to the novice if the authors were to point out the basics that are skipped over here: E.g., for the values of the bifurcation parameter where there are three steady states, the upper and lower states are stable and the middle state is unstable. And when the bifurcation parameter exceeds a critical value (at the saddle-node bifurcation), the system becomes monostable—there is a single steady-state, and it is stable. And so if CycE (or Cdc20) is changing slowly, the trajectory will cling to one leg of the S-shaped curve for as long as it can, and then plummet down (for the CycE bifurcation diagram) or explode up (for Cdc20) to the other leg.
We have expanded the section of 'Mapping the Cell Cycle Clock with Bifurcation Curves' accordingly in line 195-211.

<u>Second decision letter</u>

MS ID#: JOCES/2023/261364

© 2024. Published by The Company of Biologists under the terms of the Creative Commons Attribution License (https://creativecommons.org/licenses/by/4.0/).

MS TITLE: The oscillation of mitotic kinase governs cell cycle latches in mammalian cells

AUTHORS: Calin-Mihai Dragoi, Ekjot Kaur, Alexis R Barr, John J Tyson, and Bela Novak

ARTICLE TYPE: Research Article

I am happy to tell you that your manuscript has been accepted for publication in Journal of Cell Science, pending standard ethics checks.

Reviewer 1

Advance summary and potential significance to field

The authors have revised their paper to address my comments and I recommend publication.

Comments for the author

N/A

Reviewer 2

Advance summary and potential significance to field

A nice combination of modeling and live cell video microscopy tests some ideas about how endocycles and genome doubling can arise.

Comments for the author

The authors have done a good job of responding to my suggestions. The nice new Fig 4D is much higher in quality than what it replaces, and it gives me confidence that the authors' assessments of the timing of the start and end of S phase are valid. The movies are quite high quality too. And the textual revisions help clarify the basic logic of the modeling. I recommend publication without revision.

© 2024. Published by The Company of Biologists under the terms of the Creative Commons Attribution License (https://creativecommons.org/licenses/by/4.0/).
